# Cationic Ir(III) Complexes with 4-Fluoro-4′-pyrazolyl-(1,1′-biphenyl)-2-carbonitrile as the Cyclometalating Ligand: Synthesis, Characterizations, and Application to Ultrahigh-Efficiency Light-Emitting Electrochemical Cells

**DOI:** 10.1021/acs.inorgchem.3c03517

**Published:** 2024-03-06

**Authors:** Rong-Huei Yi, Yi-Hsun Lee, Yu-Ting Huang, Xuan-Jun Chen, Yun-Xin Wang, Dian Luo, Chin-Wei Lu, Hai-Ching Su

**Affiliations:** † Department of Applied Chemistry, Providence University, Taichung 43301, Taiwan; ‡ Institute of Lighting and Energy Photonics, National Yang Ming Chiao Tung University, Tainan 71150, Taiwan

## Abstract

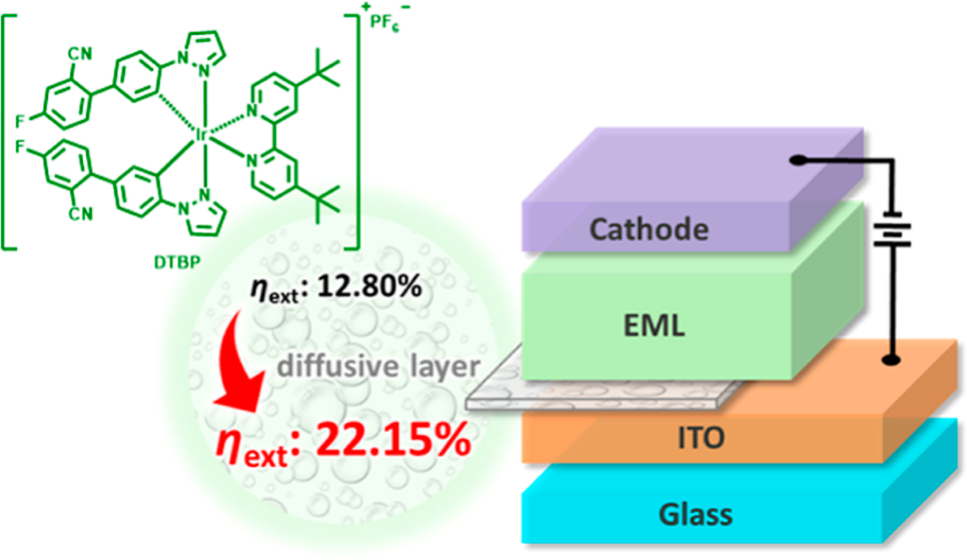

Light-emitting electrochemical cells (LECs) promise low-cost, large-area luminescence applications with air-stabilized electrodes and a versatile fabrication that enables the use of solution processes. Nevertheless, the commercialization of LECs is still encountering many obstacles, such as low electroluminescence (EL) efficiencies of the ionic materials. In this paper, we propose five blue to yellow ionic Ir complexes possessing 4-fluoro-4′-pyrazolyl-(1,1′-biphenyl)-2-carbonitrile (**ppfn**) as a novel cyclometalating ligand and use them in LECs. In particular, the device within di[4-fluoro-4′-pyrazolyl-(1,1′-biphenyl)-2-carbonitrile]-4,4′-di-*tert*-butyl-2,2′-bipyridyl iridium(III) hexafluorophosphate **(DTBP)** shows a remarkable photoluminescence quantum yield (PLQY) of 70%, and by adjusting the emissive-layer thickness, the maximal external quantum efficiency (EQE) reaches 22.15% at 532 nm under the thickness of 0.51 μm, showing the state-of-the-art value for the reported blue-green LECs.

## Abstract 2

A novel iridium complex, **DTBP**, exhibiting an exceptional PLQY of 70%, was successfully synthesized. When utilized as the emitter in LECs and with meticulous optimization of the EML thickness, it demonstrated a noteworthy EQE of 12.8%. Moreover, through the incorporation of a diffusive layer, the EQE surged to 22.15% at 532 nm, thus establishing a new benchmark for reported EQE in the domain of blue-green LECs.

## Introduction

Nowadays, the organic light-emitting diode (OLED) in display technology has afforded great success, and the vivid colors of the OLED it produces enrich our daily life. Thanks to the complexity of device fabrication that increases the energy consumed and exacerbates the energy shortage on earth, a feasible strategy is to develop the solid-sate light-emitting electrochemical cells (LECs) which are considered to be simple light-emitting devices, consisting of two electrodes and an ionic active layer (∼100 nm) sandwiched between them.^1^ The cations and anions in the emissive layer (EML) drifting and accumulating under a bias render the LECs to realize low-voltage operation.^1−5^ Although the mechanism of photon generation is similar to OLEDs, LECs still possess decisive advantages, such as simple device structures, low work function metals (e.g., aluminum or gold), and easy solution-based fabrication that make them promising light-emitting technologies for novel and inexpensive lighting and display applications.^6^ Recently, several materials have been applied to LECs with promising performance. In particular, the structure of phosphorescent ionic transition metal complexes (iTMCs) employed by iridium (Ir) metal, which exhibits a heavy atomic effect giving strong spin–orbit coupling (SOC), promotes singlet–triplet transition and achieves 100% exciton utilization.^7−17^ Besides, the σ-robust donating ability of carbon atom subtle couples with the heavy atom leading to enhance the SOC effect which can promote the exciton transition to low-lying metal-to-ligand charge transfer state (MLCT), and subsequently yield light emission through the radiative process at the excited triplet state as the El-Sayed rule.^7−13^ More importantly, the introduction of a rigid ligand can improve high phosphorescence quantum yields (QYs) leading to realize the high efficiency of LECs device.^7−13,18,19^ With these advantages, cationic Ir(III) complexes have found extensive applications in various aspects of LECs.

Primarily, cationic cyclometalated Ir(III) complexes are typically represented by [Ir(C∧N)_2_(N∧N)]^+^PF_6_^–^, with the C∧N ligand serving as the cyclometalating ligand and the N∧N ligand as an ancillary one. Consequently, these complexes exhibit both MLCT and ligand-to-ligand charge transfer (LLCT) characteristics. By carefully selecting appropriate ligands, it becomes possible to achieve high luminance efficiency and create multicolor LECs.^7,9,13,22−24^ To obtain emission at 450–650 nm, one approach consists of using either electron-withdrawing group-substituted ligands (such as F) or electron donating group-substituted ligands (such as CH_3_ or OCH_3_) showing a high-lying lowest-unoccupied molecular orbital (LUMO) while azole-containing moieties as the highest-occupied molecular orbital (HOMO).^25−29^ It was recently shown that the replacement of the pyridine ring of C∧N ligand by the five-membered nitrogen-rich heterocyclic moiety, e.g., pyrazole,^30^ triazole,^31^ or tetrazole,^32^ gives in most cases a hypsochromically shifted PL emission due to the HOMO stabilization.^33^ According our previous work, tethering electron-withdrawing fluorine atoms onto phenylpyrazole and introducing electron-donating CH_3_ moieties onto bipyridine (bpy) lead to an enhanced band gap (*E*_g_) and excellent performance of a maximal external quantum efficiency (EQE_max_) = 4.6% in blue-green LECs.^30,34−36^ In 2020, Lu et al. successfully developed a yellow complex known as YIr, denoted as [Ir(bppz)_2_(Bphen)]PF_6_, using the host–guest strategy and embedding a diffusive layer, achieving a promising EQE_max_ up to 23.7% at 565 nm, marking it as the best performance observed to date for yellow LECs.^20^ In the subsequent year, a green complex, [Ir(CF_3_-dPhTAZ)_2_(bpy)]PF_6_, which introduced by He et al. achieved an EQ*E*_max_ of 10.4% at 525 nm while effectively mitigating phosphorescence concentration-quenching using the C∧N ligand.^21^

To further boost the device performance of LECs, enhancing the photoluminescence QY (PLQY) of Ir(III) complexes is a practical strategy. Lately, Choe et al. developed novel complexes chelated with phenanthroimidazole typed C∧N ligand which fuse a phenyl ring, resulting in more rigid structure and longer π-conjugation length that can suppress the nonradiative process and intermolecular π–π interactions.^37^ Hence, the incorporation of a phenyl π-bridge into the difluorophenylpyrazole ligand serves to not only extend π-conjugation but also increase steric hindrance, which proves advantageous for radiative deactivation in excited states. Additionally, the effect of enhancing the polarity of the C∧N ligand through the substitution of fluorine with a cyano group remains unexplored in the context of LECs.

In this work, 4-fluoro-4′-(1*H*-pyrazol-1-yl)-[1,1′-biphenyl]-2-carbonitrile (**ppfn**) was chosen to control the C∧N ligand while introducing the benzene ring increases the solubility, and this strategy speculated that the charge transport can be improved. Herein, several blue to yellow cyclometalated iridium complexes with different substituents and heterocycles of N∧N ligands are synthesized. Among them, the device using di[4-fluoro-4′-pyrazolyl-(1,1′-biphenyl)-2-carbonitrile]-4,4′-di-*tert*-butyl-2,2′-bipyridyl iridium(III) hexafluorophosphate (**DTBP**) performs remarkably PLQY of 70% and by emissive-layer optimization, a high EQE_max_ even reaches 22.15% at 532 nm under the thickness of 0.51 μm, showing the best value for the published blue–green LECs.

## Experimental Section

### Materials and Synthesis

Detailed synthesis procedures are shown in the Supporting Information.

### Fabrication of LECs and EL Measurements

Cleaning and ultraviolet (UV)/ozone processing of indium tin oxide (ITO)/glass substrates were carried out before device fabrication. After that, poly(3,4-ethylenedioxythiophene):poly(styrenesulfonate) (PEDOT:PSS) layers with thickness of *ca*. 40 nm were deposited by spin coating (4000 rpm) onto the ITO/glass substrates. They were then heated at 150 °C for half an hour. The ionic liquid 1-butyl-3-methylimidazolium hexafluorophosphate [BMIM^+^(PF_6_)^−^] (20 wt %) was included in EML to offer additional ions to reduce the response time of device. All EMLs of *ca*. 215 nm were deposited by spin coating (3000 rpm) from a complex/[BMIM^+^(PF_6_)^−^] mixture in MeCN (80 mg mL^–1^). To optimize the device performance of LECs based on complex **DTBP**, various MeCN solutions with concentrations of 60, 80, 100, 120, 160, and 200 mg mL^–1^ were used to fabricate EMLs with thicknesses of 169, 215, 264, 299, 413, and 507 nm, respectively. EML thickness was determined with ellipsometry. After EML deposition, they were heated at 70 °C for 10 h. Eventually, Ag cathodes were thermally evaporated on top of EML. To enhance the light extraction from LECs based on complex **DTBP**, the optimized device (EML thickness 507 nm) was fabricated on the diffusive substrate containing a diffuser film between the ITO layer and glass substrate. A transparent photoresist layer mixed with TiO_2_ nanoparticles served as the diffuser film and the detailed fabrication processes have been reported in previous literature.^20,38^ These devices’ EL characteristics were obtained with a silicon photodiode and source-measurement units (Keysight B2900A). The fiber optic spectrometer (USB4000) was utilized to measure EL spectra of these devices. To keep these devices from quick degradation, constant-current EL measurements were performed in a glovebox filled with inert nitrogen.

## Results and Discussion

### Synthesis and Structural Characterization

**Scheme 1:**
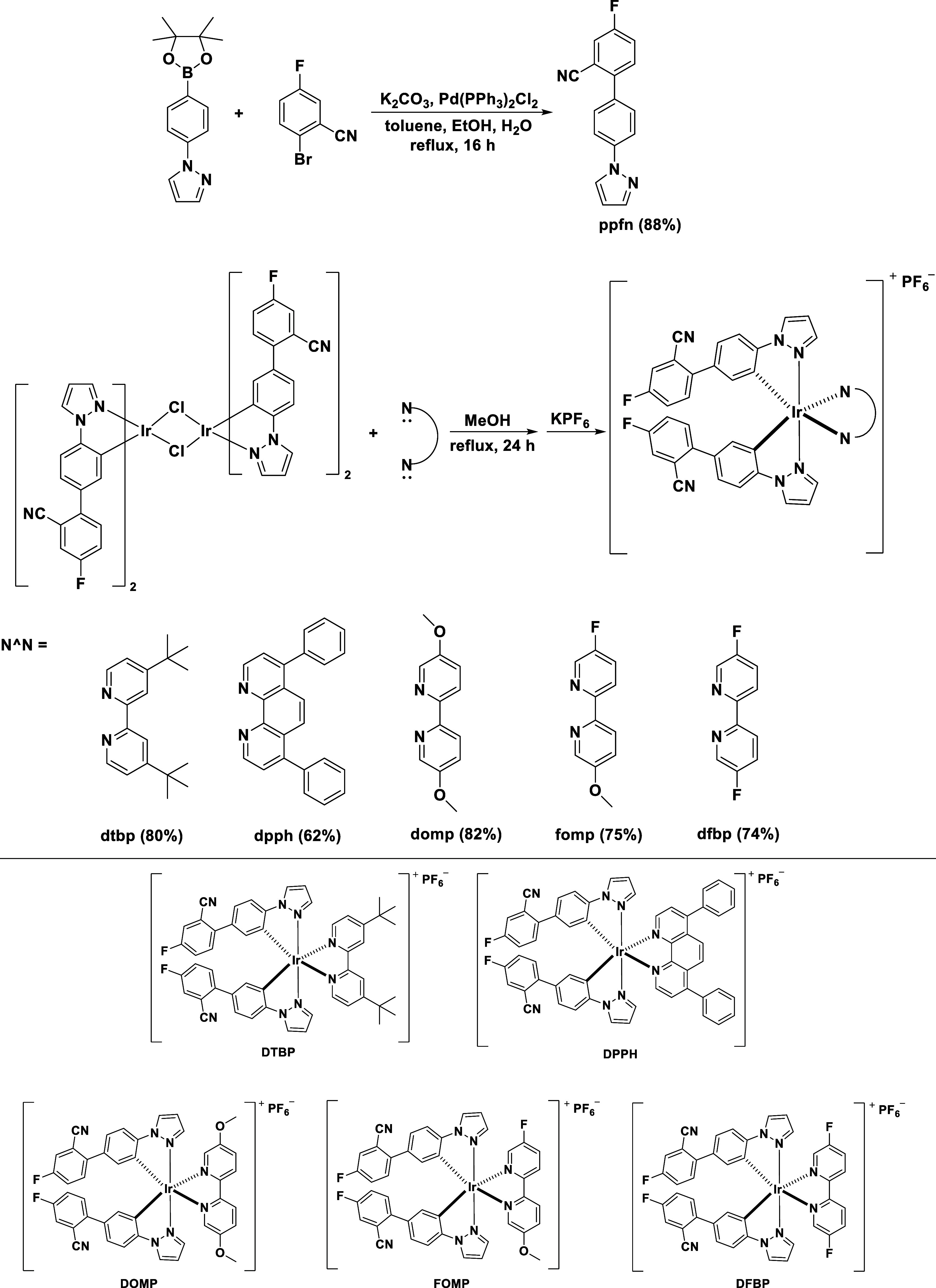
Synthesis of C∧N Ligand (**ppfn**) and all Targeted Complexes

Scheme 1 shows the synthetic route to ligand **ppfn** and targeted complexes **DTBP**–**DFBP**. First, the pyrazole-based ligand was synthesized via Suzuki coupling and then reacted with IrCl_3_·3H_2_O to obtain the chloro-bridged Ir dimer **[Ir(ppfn)**_**2**_**Cl]**_**2**_. Afterward, the reaction exhibiting various electron-push–pull pyridine-based N∧N ligands (**dtbp**, **dpph**, **domp**, **fomp**, and **dfbp**(39)) and anion–exchange reactions from Cl^–^ to PF_6_^–^ yielded complexes **DTBP**–**DFBP**. All of the targeted complexes were purified with silica gel column chromatography. Then, they were recrystallized in a dichloromethane (DCM)/*n*-hexane mixed solution. All of them are highly soluble in polar solvents, e.g., DCM, MeCN, and DMSO. However, they show a low solubility in nonpolar solvents. These Ir(III) complexes were identified by ^1^H NMR, ^13^C NMR, single crystal XRD, elemental analysis, and high-resolution mass spectrometry. Characterization data of these complexes are included in the Supporting Information (PDF).

**Figure 1:**
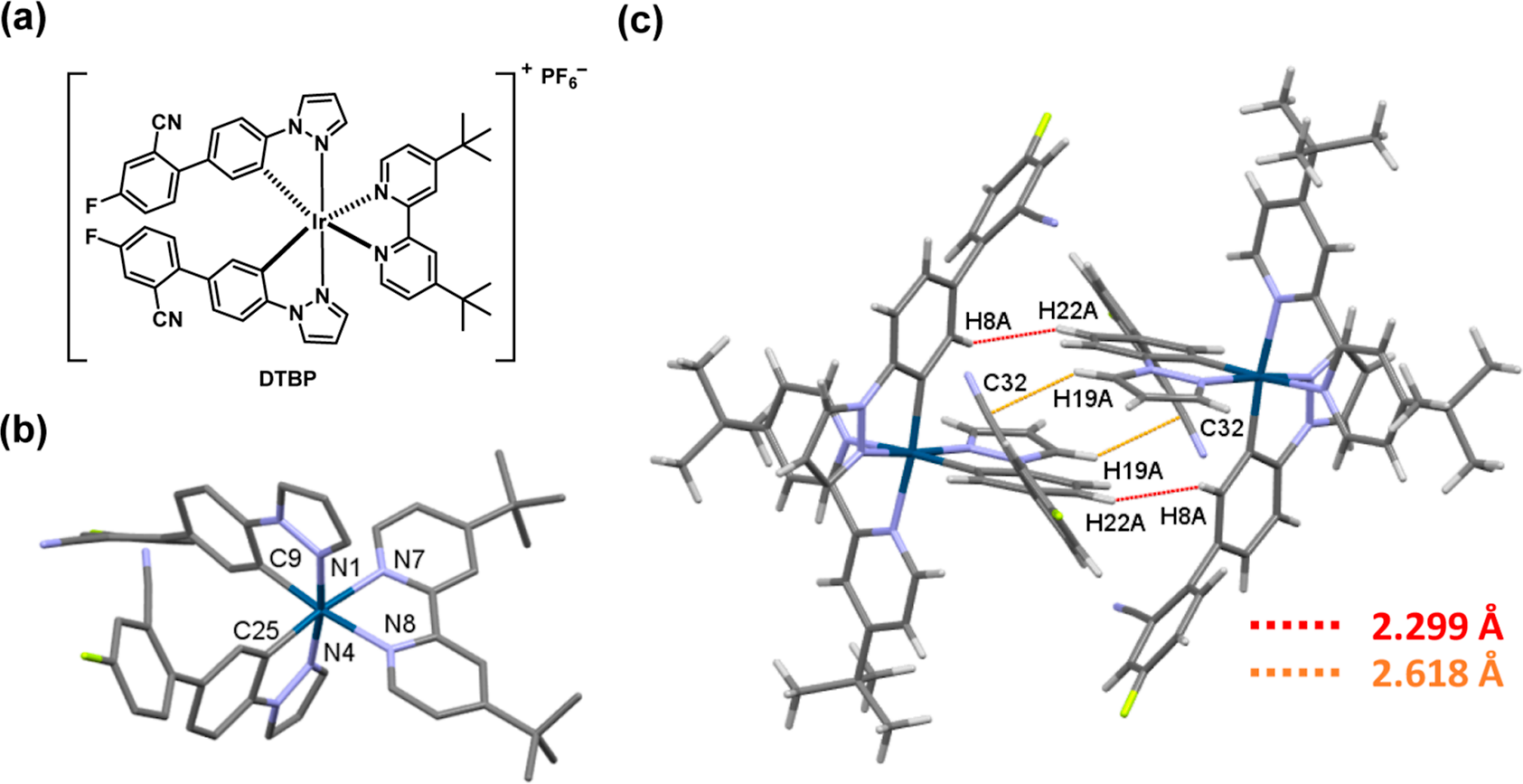
(a) Molecular and (b) single crystal structure of **DTBP** and (c) intermolecular interactions in **DTBP** crystal. PF_6_^–^ counterion is omitted.

The molecular structure and crystal packing of complex **DTBP** was characterized with the single-crystal X-ray diffraction (Figure 1). By employing chloroform and *n*-hexane, the single crystal was prepared with a double-layer diffused recrystallization. The crystal data are shown in Table S1. Obviously, the complex presents a pseudooctahedral geometry containing Ir as the metal center. The periphery consists of three bidentate ligands. Two of them are C∧N ligands, while the other one is a N∧N ligand. It is a common cyclometalated iridium complex {[Ir(C∧N)_2_(N∧N)]^+^}. Complex **DTBP** is crystallized in the triclinic *P*-1 space group. In structure, the bond lengths for the Ir-ligand in the complex in Å are Ir–C9 = 2.013(3), Ir–N4 = 2.017(3), Ir–N1 = 2.024(3), Ir–C25 = 2.028(4), Ir–N7 = 2.117(3), and Ir–N8 = 2.124(3), respectively. All of these values fall into the expected ranges. Furthermore, complex **DTBP** demonstrates that intermolecular interactions originate from the C∧N ligands (i.e., **ppfn**) interacting with each other, as illustrated in Figure 1c. These interactions are characterized by distances of 2.618 Å for C32–H19 and 2.299 Å for H8–H22, respectively, signifying a strong intermolecular interaction within this complex.

### Photophysical Characteristics

**Figure 2:**
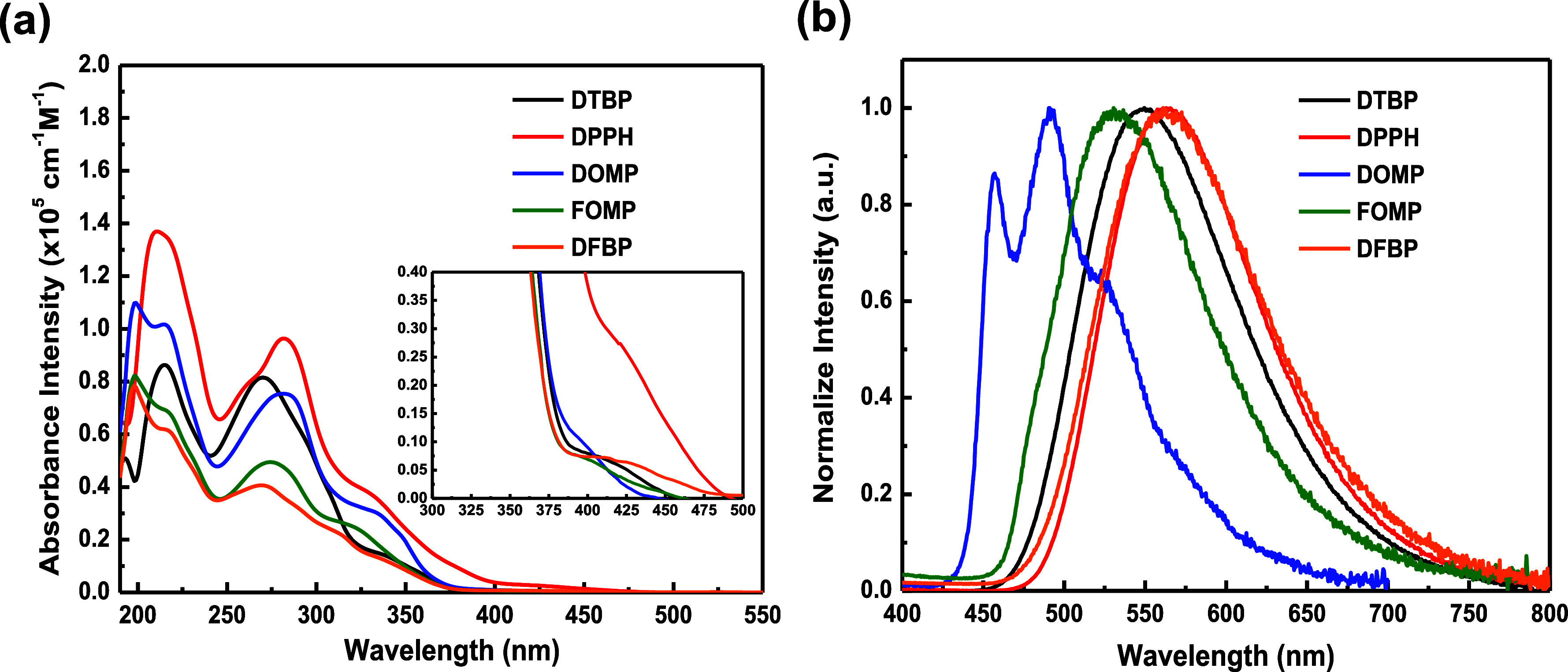
(a) Absorption and (b) PL spectra of complexes in MeCN (1.0 × 10^–5^ M).

Absorption and PL spectra for complexes **DTBP**–**DFBP** (MeCN solution) are depicted in Figure 2. Table 1 summarizes the corresponding photophysical data. For all complexes, the absorption peaks locate within 200–300 nm, which are attributed to π–π* transition from ligands. Longer-wavelength absorption (300–350 nm) comes from spin-allowed MLCT and LLCT, while weaker absorption bands (>400 nm) result from the spin-prohibited MLCT, LLCT, and ligand centered (LC) transitions.^40^ Interestingly, **DPPH** exhibits stronger extinction coefficient (ε > 10^4^ M^–1^ cm^–1^) due to the high oscillator strength resulting from the highly rigid structure of **dpph**.

**Table 1:** Summary of the Physical Properties of Targeted Complexes

complex	absorption^a^ [nm]	emission^a^ [nm]	Φ^b^/Φ^c^ [%]	τ_obs_^d^/τ_obs_^e^ [μs]	*E*_1/2_^ox^^f^ [V]	*E*_1/2_^red^^f^ [V]	HOMO [eV]	LUMO [eV]	*k*_r_^g^ [10^5^ s^–1^]	*k*_nr_^g^ [10^5^ s^–1^]
**DTBP**	215,270,338	550	70/60	0.741/0.778	1.13	–0.99	–5.82	–3.73	9.54	4.05
**DPPH**	210,262,283,335	563	48/46	1.406/1.542	1.10	–1.02	–5.94	–3.70	3.41	3.70
**DOMP**	198,262,283,337	456,491,530	46/46	1.467/1.623	1.06	–1.00	–5.86	–3.72	3.14	3.68
**FOMP**	198,218,275,325	531	58/56	0.897/0.879	1.14	–0.97	–5.90	–3.75	6.47	4.68
**DFBP**	198,218,271,340	561	42/41	0.603/0.813	1.02	–0.92	–5.93	–3.81	6.97	9.62

a Measured in 1 × 10^–5^ M MeCN.

b Absolute PLQY of neat film measured by using an integrating sphere.

c Absolute PLQY of the thin-film contained complex (80 wt %) and [BMIM^+^(PF_6_)^−^] (20 wt %) measured by employing an integrating sphere.

d Excited-state lifetime for neat film.

e Excited-state lifetime for thin film contained complex (80 wt %) and [BMIM^+^(PF_6_)^−^] (20 wt %).

f Using TBAPF_6_ as the electrolyte (0.1 M in MeCN). Potential vs ferrocene/ferrocenium redox couple.

g Radiative decay rate constants (*k*_r_) and nonradiative decay rate constants (*k*_nr_) were derived following the equations *k*_r_ = Φ × τ^–1^ and *k*_nr_ = (1 – Φ) × τ^–1^, respectively. Φ and τ are neat-film PLQY and excited-state lifetime, respectively.^41^

These complexes were measured in MeCN solution for PL emission with wide spectra, except **DOMP**. The emission of all complexes shows the red-shifted with increasing the electron-withdrawing ability. Among them, **DOMP** demonstrated significant fine-structured spectra implying **domp** which have weaker electron-withdrawing ability, coupling with **ppfn** leading to the lying locally excited (LE) state emission. For **DPPH**, the longer π-conjunction of **dpph** promotes the electron-withdrawing ability and further induces obvious MLCT emission. Notably, **DTBP** exhibits slightly hypsochromic shifted emission coming from the enhanced electron-donating ability of **dtbp** compared to **DOMP**, and this part can be identified from the distribution of the HOMO and LUMO through density functional theory (DFT) results. To deeply realized the photophysical properties of complexes, the transient PL (TrPL) measurement are probed. Figure S14 depicts the TrPL spectra of all complexes. Table 1 summarizes the data of the excited-state lifetimes obtained from the transient PL profiles. The excited-state lifetime can be fitted by a single-exponential decay of 0.741–1.406 μs in the neat film, confirming the phosphorescent characteristic. The PLQYs of **DTBP**–**DFBP** measured in an integration sphere are 70, 48, 46, 58, and 42%, respectively. To realize the structure–property relationship with the dynamic processes of the excited states, radiative and nonradiative decay rates (*k*_r_ & *k*_nr_) of complexes **DTBP**–**DFBP** were estimated with the following equations: *k*_r_ = Φ/τ and *k*_nr_ = (1 – Φ)/τ, where τ is the excited-state lifetime and Φ is the measured PLQY.^41^ Such values are typical for metal complexes with a very strong SOC involving a mixture of the main MLCT excited state and a large number of d orbitals from the metal and are usually confined to Ir(III) based emitters, such as a commercialized *fac*-Ir(ppy)_3_ with a *k*_r_ of about 5 × 10^5^ s^–1^.^42^ The *k*_r_ and *k*_nr_ of **DTBP** can be calculated to be 9.54 × 10^5^ and 4.05 × 10^5^ s^–1^, respectively. This result demonstrates the utilization of C∧N ligand **ppfn** with the *tert*-butyl group on N∧N ligand **dtbp**, reducing intermolecular interactions and suppressing nonradiative processes, while exhibiting slight *J*-aggregation (Figure 1c), leading to a higher *k*_r_.^43^

### Electrochemical Properties and Theoretical Calculations

**Figure 3:**
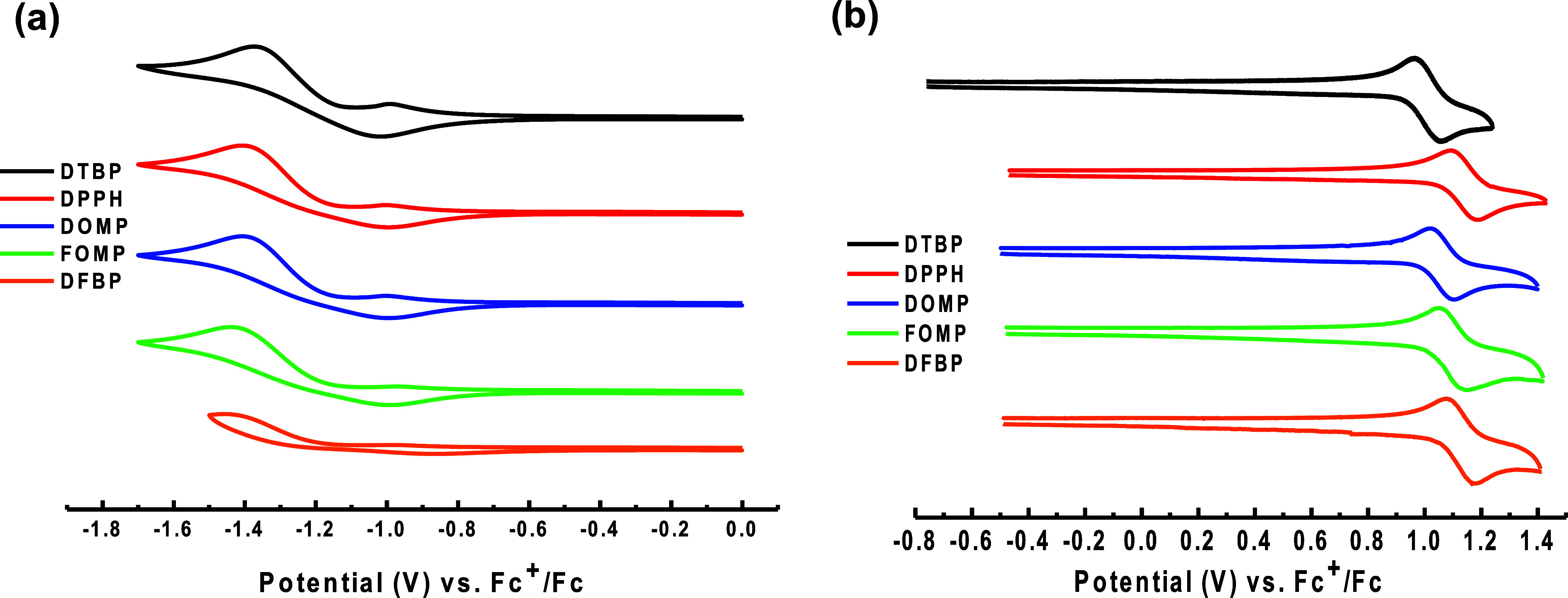
Cyclic voltammograms of (a) reduction and (b) oxidation processes of the targeted complexes in MeCN. Potentials were recorded vs Fc^+^/Fc.

To shed light on the electrochemical characteristics, the cyclic voltammetry (CV) of these complexes were carried out in MeCN. The data are summarized in Table 1. Complexes **DTBP**–**DFBP** all exhibit a reversible oxidation process in Figure 3. Among them, **DTBP** contains the strongest electron-donating group (**dtbp**), leading to the lowest *E*_1/2_^ox^ at 1.02 V compared to other complexes. Interestingly, the earliest oxidation process of **DOMP** proceeds at 1.06 V compared to that of **FOMP** (1.10 V) and **DFBP** (1.13 V) due to the modification of bipyridine at the *meta*-position with methoxyl, which is a strong donor. For **DPPH**, **dpph** has deficient electronic properties and shows a deeper *E*_1/2_^ox^ of 1.14 V. The results show that the different electronic characteristics of the N∧N ligands are involved in the oxidation process of these complexes. Refer to the redox couple of ferrocenium/ferrocene (Fc^+^/Fc),^44,45^ the HOMO levels of **DTBP**, **DPPH**, **DOMP**, **FOMP**, and **DFBP** are calculated as −5.82, −5.94, −5.86, −5.90, and −5.93 eV, respectively. After that, the HOMO levels add up the optical band gap *E*_g_, which derived from the onset of absorption, giving the corresponding LUMO levels of −3.10, −3.39, −3.42, −3.06, and −3.22 eV for **DTBP**, **DPPH**, **DOMP**, **FOMP**, and **DFBP**, respectively.

To understand the structure–property correlation, DFT calculations of **DTBP**–**DFBP** were conducted. The optimized molecular structure of the ground state at a B3LYP/LANL2DZ level is shown in Figure S16. Figure S17 depicts the calculated data of the HOMO and LUMO distributions. Little difference on the HOMO distributions for these five complexes is found owing to their similar structures. These complexes’ HOMOs are contributed from the metal center and extend to the C∧N ligand (**ppfn**). Notably, the N∧N ligands are also coupled to the HOMO distribution as the electron-donating ability of the N∧N ligand increases. It is in line with the CV data. LUMOs of **DFBP**, **FOMP**, and **DFBP** mainly lie on the whole N∧N ligands. This result agrees well with our previous publication.^20^ Particularly, the LUMOs of **DFBP** and **DOMP** are distributed on the 3-fluorobenzonitrile due to modification of the N∧N ligands by *tert*-butyl and methoxyl groups. Obviously, the subtle separately distribution of HOMO and LUMO implies that the fast MLCT process dominates at low-lying excited state which is beneficial with down-conversion process of singlet-to triplet transition and radiative deactivation.

Time-dependent DFT (TD-DFT) utilizing m06-2*x*/6-31G(d)/LANL2DZ, a hybrid meta-generalized gradient approximation functional,^46^ was performed to optimize the T_1_ structure (Figure S18). Table S2 summarizes the emission peak wavelengths calculated from the TD-DFT simulation results. The natural transition orbital (NTO) describing the T_1_ to S_0_ transition between the excited particle and empty hole is depicted in Figure S19. Since the more electron-donating ability of methoxyl than fluorine, the intensity arrangement of electron-donating ability is **domp**, **fomp**, and **dfbp**, respectively. Therefore, the distribution of the hole is mainly distributed on the N∧N ligand (**domp**) in **DOMP**, while the distribution of **FOMP** is delocalized at the C∧N ligand (**ppfn**) and slightly coupled with the N∧N ligand (**fomp**). For **DFBP**, the distribution of hole is in a degenerate state, which is delocalized between two C∧N ligands. The distribution of particles in the three complexes is distributed on the N∧N ligands (**domp**, **fomp**, and **dfbp**, respectively). These results reveal that the radiative transition of **DOMP** exhibits ^3^LE characteristics, leading to a fine-structured profile of emission. In **DPPH**, the distribution of the hole and particle is similar to **FOMP**. As the substitution of 4 and 7 positions for **dpph** can increase the electron-donating ability, resulting in extended p-conjugation length, the hole contribution consisted of C∧N and N∧N ligands. Interestingly, the distribution of hole and particle for **DTBP** are distributed on the C∧N ligand, which is speculated to have both electron-donating and electron-withdrawing character in the whole ligand skeleton. The result show that the MLCT process can proceed from the same ligand with intramolecular charge-transfer characteristic, combining the heavy-atom effect of Ir to giving the fast ISC and radiative process. This is the prerequisite for a spin-flip transition to be allowed by El-Sayed’s rule.

### Electroluminescent Properties of LECs

**Figure 4:**
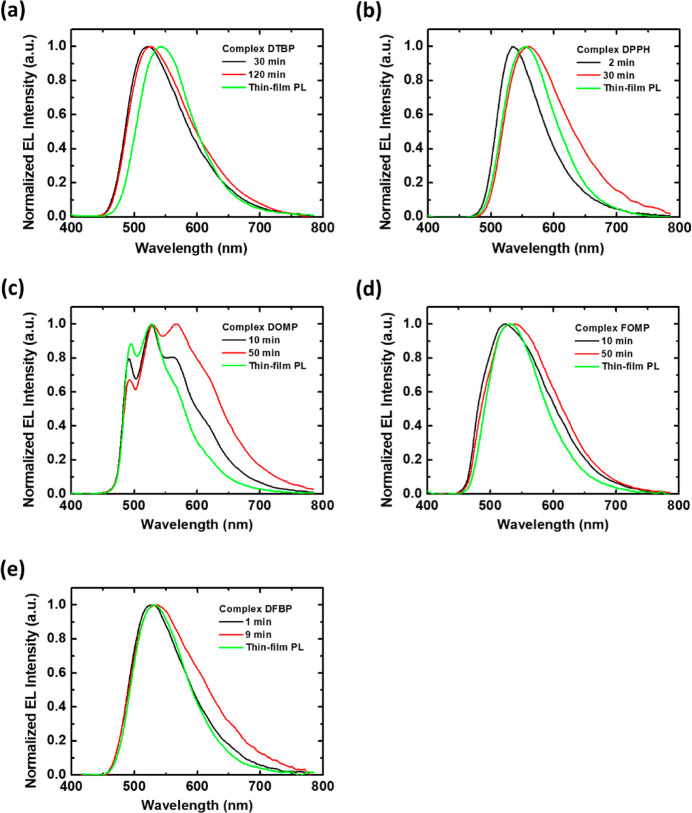
Initial and steady-state EL spectra for the LECs (*ca*. 215 nm) using (a) **DTBP**, (b) **DPPH**, (c) **DOMP**, (d) **FOMP**, and (e) **DFBP** driven under currents of 0.1, 5, 1, 0.25, and 5 μA, respectively. The thin-film PL spectra of EML with complex (80 wt %) and [BMIM^+^(PF_6_)^−^] (20 wt %) are incorporated to facilitate comparison.

The LECs based on these complexes were tested to examine their EL characteristics. Table 2 summarizes the EL properties. The EMLs of five iTMCs were deposited by spin coating from the complex/[BMIM^+^(PF_6_)^−^] mixture (80 mg mL^–1^). MeCN was used as the solvent in the spin-coating of the EMLs of all complexes. The EML thicknesses of these devices were measure to be *ca*. 215 nm. All EL spectra of the LECs based on these complexes are shown in Figure 4. All devices showed temporal evolution of EL spectra since moving emission zone during LEC operation altered microcavity effect and changed the output EL spectra with time.^47−49^ Thin-film PL spectra of the EML contained complex (80 wt %) and [BMIM^+^(PF_6_)^−^] (20 wt %) are incorporated in Figure 4 to facilitate comparison. The EL spectra of these iTMCs resembled their PL spectra, despite time-dependent and altered EL spectra modified by optical interference. It implies similar PL and EL emission mechanisms for these iTMCs. As such, these iTMCs indeed work well in LEC devices.

**Table 2:** Summary of EL Properties of the Proposed Complexes (*ca*. 215 nm)

complex	current [μA]	E*L*_max_^a^ [nm]	CIE^b^ [*x*, *y*]	*B*_max_^c^ [cd m^–2^]	η_ext, max_^d^ [%]	η_c, max_^e^ [cd A^–1^]	η_P, max_^f^ [lm W^1–^]
**DTBP**	0.1	522 to 527	(0.34, 0.55) to (0.35, 0.55)	3.08	10.30	30.78	36.25
**DPPH**	5	534 to 560	(0.37, 0.59) to (0.45, 0.53)	155.22	9.24	31.04	31.15
**DOMP**	1	492, 527, 567, 618 (sh.)	(0.36, 0.54) to (0.41, 0.52)	13.70	4.64	13.70	14.57
**FOMP**	0.25	523 to 538	(0.35, 0.54) to (0.37, 0.54)	5.57	7.71	22.30	24.84
**DFBP**	5	530 to 535	(0.35, 0.56) to (0.38, 0.52)	13.60	0.93	2.72	2.69

a EL peak wavelength.

b CIE 1931 coordinate.

c Maximal brightness.

d Maximal external quantum efficiency.

e Maximal current efficiency.

f Maximal power efficiency.

**Figure 5:**
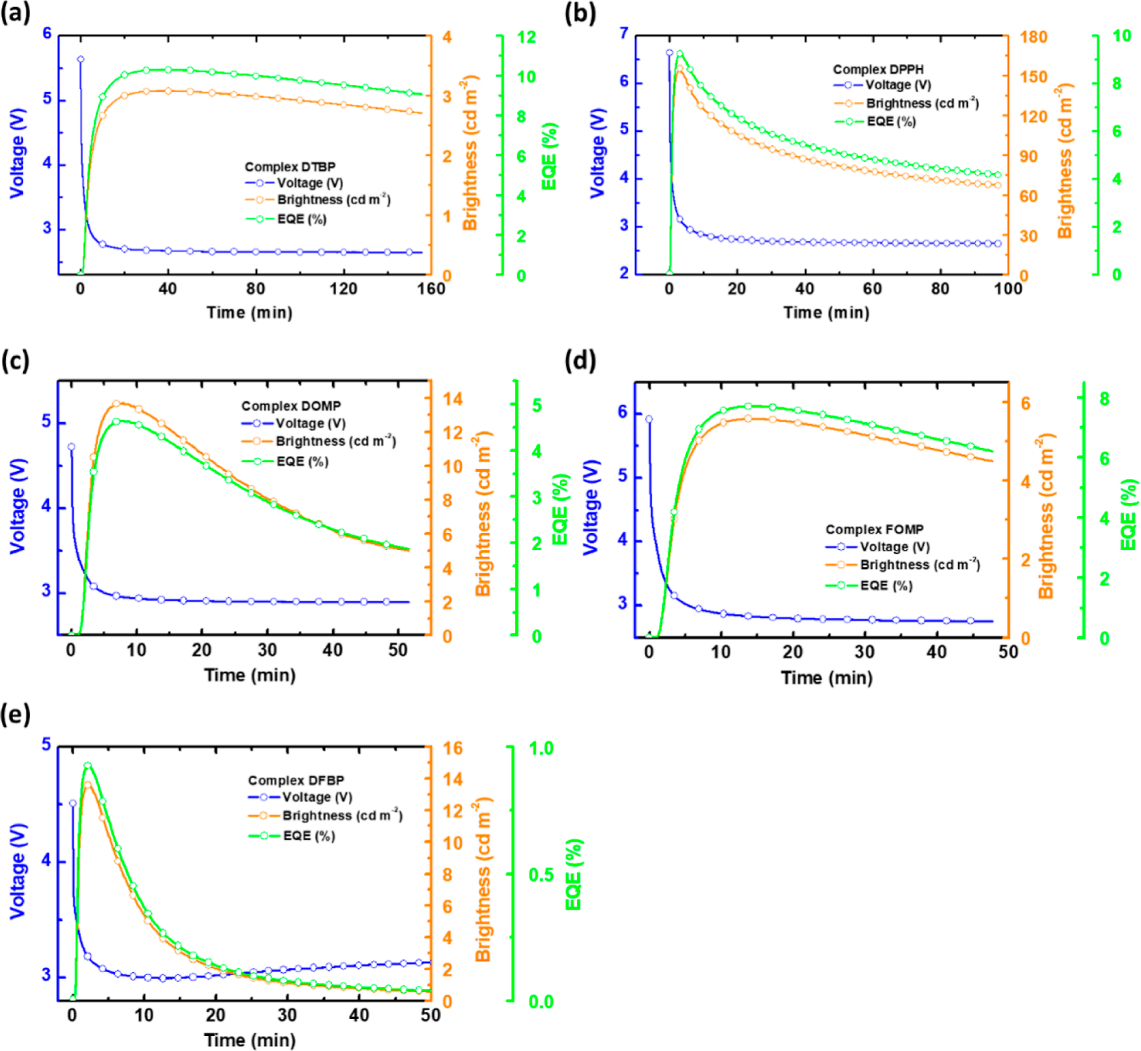
Time-dependent voltage (blue lines), brightness (orange lines), and EQE (green lines) of the LECs (*ca*. 215 nm) based on complexes (a) **DTBP**, (b) **DPPH**, (c) **DOMP**, (d) **FOMP**, and (e) **DFBP** driven by constant currents of 0.1, 5, 1, 0.25, and 5 μA, respectively.

The temporally evolved voltages (blue lines) of the LECs utilizing the proposed iTMCs driven by constant currents are shown in Figure 5. The selected current employed for an individual LEC was optimized for the best device EQE. All LECs showed a similar temporal trend in device voltages when driven under constant currents. At the beginning, the conductively doped layers have not established completely, and thus, a relatively higher bias was required to overcome high carrier injection barriers at the electrodes. When the conductively doped layers grew up gradually, the required device voltage to reach the target current decreased with time owing to lowered carrier injection barriers. In the end, the device voltage remained constant after the conductively doped layers were stabilized. The temporally evolved brightness (orange lines) and EQE (green lines) of the LECs using the proposed iTMCs under constant currents are also shown in Figure 5. The carrier balance significantly improved, owing to the growing conductively doped layers such that the brightness increased gradually. When the brightness passed the maximal point, the brightness decreased with time. It may be related to exciton quenching in the proximity of growing conductively doped layers and irreversible material aging.^50,51^ The temporally evolved EQE followed a similar temporal trajectory of the brightness due to constant-current operation. The LEC based on complex **DTBP** showed the highest peak EQE (10.23%) among all devices. When considering the thin-film contained complex (80 wt %) and [BMIM^+^(PF_6_)^−^] (20 wt %) PLQY of complex **DTBP** (60%, Table 1) and *ca*. 20% light outcoupling efficiency, such a high EQE from the LEC based on the proposed green iTMC with **dtbp** ligand reached *ca*. 85% of the theoretical maximum value. The device employing complex **DPPH** also delivered a good EQE of 9.24%. Slightly lower EQE compared with the LEC based on **DTBP** resulted from the lower PLQY of complex **DPPH** (46%, Table 1). Among the LECs employing complexes **DOMP**, **FOMP**, and **DFBP**, the LEC based on complex **FOMP** showed the best peak EQE (7.71%), while a reduced peak EQE (4.64%) was obtained from the device using complex **DOMP** and the device utilizing complex **DFBP** exhibited the worst peak EQE (0.93%). Considering the PLQYs of their thin films contained complex (80 wt %) and [BMIM^+^(PF_6_)^−^] (20 wt %) (Table 1) and light outcoupling efficiency (*ca*. 20%), the EQEs from the LECs employing complexes **FOMP**, **DOMP**, and **DFBP** reached 69, 50, and 11%, respectively, of their theoretical maximum values. It revealed that the complex possessing the bpy ligand with an electron-withdrawing fluorine substituent and an electron-donating OCH_3_ substituent (complex **FOMP**) exhibits a higher thin-film contained complex (80 wt %) and [BMIM^+^(PF_6_)^−^] (20 wt %) PLQY and better EL property than that with two electron-donating OCH_3_ substituents (complex **DOMP**). Complex **DFBP** showed a similar thin-film contained complex (80 wt %) and [BMIM^+^(PF_6_)^−^] (20 wt %) PLQY to that of complex **DOMP**, but the LEC based on complex **DFBP** delivered a significantly lower EQE, revealing its poor EL property. It indicated that the bpy ligand with two electron-withdrawing fluorine substituents was detrimental to the EL property of the LEC based on the corresponding complex. Therefore, adjusting the electron-withdrawing or electron-donating property of the ligand is essential in optimizing the device performance of the Ir(III) complexes based LEC.

In view of the high PLQY and superior EL property of complex **DTBP**, the EML thickness of the LEC based on complex **DTBP** was tuned to optimize the device efficiency. Adjusting EML thickness can be employed to optimize EL properties for LEC devices.^20,52^ A wide range of EML thicknesses (169, 215, 264, 299, 413, and 507 nm) was examined for the LECs based on complex **DTBP** and their corresponding EL properties are listed in Table 3. EL spectra for the LECs based on EMLs of complex **DTBP** with various thicknesses under constant currents are depicted in Figure S25. The EL spectrum changed for different EML thickness, especially for thicker EML, due to optical modification from the microcavity effect resulting from different optical architectures and dissimilar location of the emission zone. The time-dependent voltages (blue lines), brightness (orange lines), and EQE (green lines) for the LECs using complex **DTBP** exhibiting different thicknesses under constant currents are depicted in Figure S25. The LECs based on complex **DTBP** with all thicknesses exhibited similar time-dependent EL properties. Nevertheless, the EQE can be enhanced by more than 200% when the EML thickness is increased from 169 to 507 nm. The optimal peak EQE of the thickest LECs based on complex **DTBP** (507 nm) reached 12.80%. It resulted from that reduced quenching effect in the proximity of electrochemically doped regions is achieved more easily for thicker LECs, in which sufficient spacing between emission zone and p- and n-type doped layers is ensured.^53^ In addition, the light outcoupling efficiency also dependents on the device thickness and thus it may enhance the outcoupled EL as well.^20^

**Figure 6:**
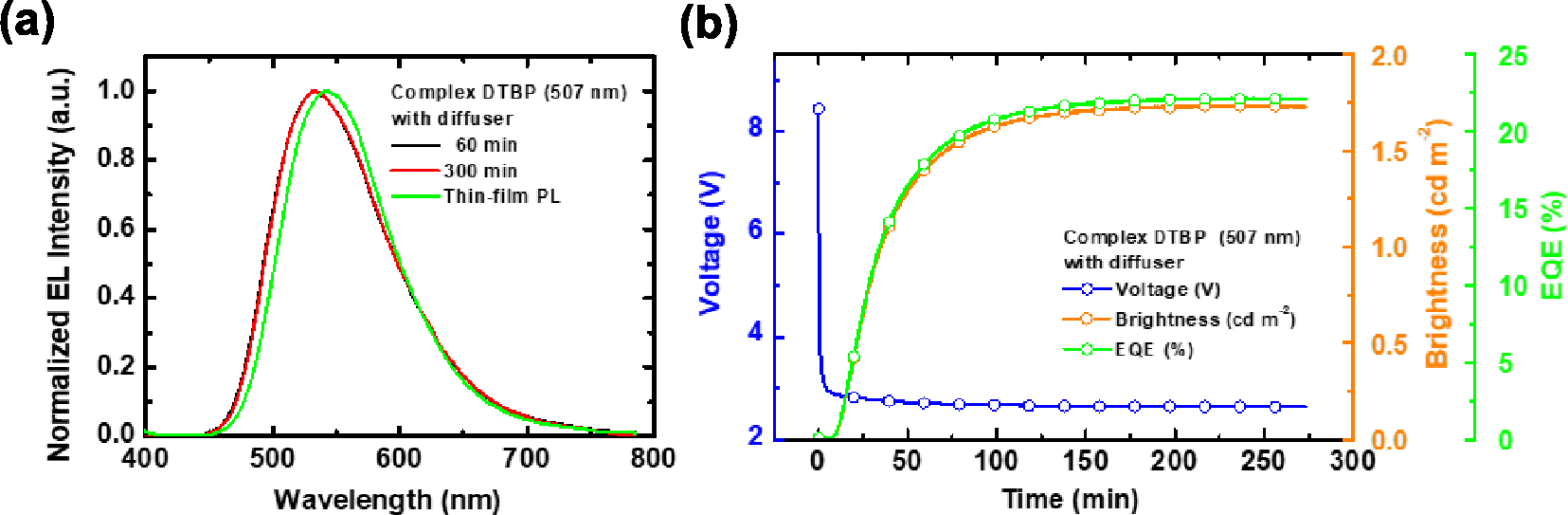
(a) EL spectra of the optimized LECs utilizing complex **DTBP** (507 nm) on diffusive substrates, operating under a constant current of 0.025 μA. A comparison with the thin-film PL spectrum of complex **DTBP** is also provided. (b) Time-dependent voltage (blue lines), brightness (orange lines), and EQE (green lines) characteristics of the LECs employing complex **DTBP** (507 nm) on diffusive substrates, with a constant current of 0.025 μA.

To further enhance the device efficiency of LECs, recycling of the light trapped in substrate and waveguide modes has been reported.^54,55^ It can be realized by inserting a diffusive layer, which is composed of a transparent photoresist layer doped with TiO2 nanoparticles, between ITO layer and glass substrate.^20,38,56^ The EL spectra of the optimized LECs based on complex **DTBP** (507 nm) fabricated on diffusive substrates (ITO/diffusive layer/glass substrates) are shown in Figure6a. It is noted that the EL spectra were almost time-independent. The diffusive substrate destroyed the microcavity effect coming from the resonant optical feedback from substrate and the EL spectrum consequently recovered the intrinsic emission spectrum of the EML. It was confirmed by the almost identical thin-film PL and EL spectra from the LECs on diffusive substrates (Figure6a). In addition, the diffusive layer redirected some of the trapped light in glass substrate and ITO waveguide into forward direction, rendering significantly enhanced light output. As shown in Figure6b and Table 3, the peak EQE and power efficiency obtained from the optimized LECs based on complex **DTBP** (507 nm) fabricated on the diffusive substrates were 22.15% and 82.87 lm W^−1^, respectively. These results are among the highest reported values from the LECs based on iTMCs with light outcoupling enhancement technique and thus confirm that the proposed complex **DTBP** shows great potential in highly efficient LECs.

**Table 3:** Summary of the Device EL Characteristics of the LECs Based on EMLs of Complex DTBP with Various Thicknesses

EML thickness [nm]	current [μA]	EL_max_^a^ [nm]	CIE^b^ [x, y]	B_max_^c^ [cd m^-2^]	η_ext, max_^d^ [%]	η_c, max_^e^ [cd A^–1^]	η_P, max_^f^ [lm W^–1^]
169	0.25	554	(0.38, 0.56)	5.21	6.21	20.85	24.30
215	0.1	522 to 527	(0.34, 0.55) to (0.35, 0.55)	3.08	10.30	30.78	36.25
264	0.25	519 to 524	(0.30, 0.60) to (0.31, 0.60)	10.12	12.13	40.50	46.23
299	0.1	559	(0.39, 0.55)	4.09	12.39	40.85	47.41
413	0.1	512 to 529	(0.29, 0.58) to (0.39, 0.55)	3.75	12.51	37.50	41.50
507	0.1	577 to 592	(0.42, 0.51) to (0.46, 0.48)	3.49	12.80	34.95	39.81
507^g^	0.025	532	(0.36, 0.56)	1.73	22.15	69.37	82.87

a EL peak wavelength of EL spectrum.

b CIE 1931 coordinate of EL spectrum.

c Maximal brightness.

d Maximal external quantum efficiency.

e Maximal current efficiency.

f Maximal power efficiency.

g With a diffuser film between ITO layer and glass substrate.

## Conclusions

We proposed a novel C∧N ligand **ppfn**, and paired it with a series of N∧N ligands **dtbp**, **dpph**, **domp**, **fomp**, and **dfbp** with different electron-withdrawing capabilities,^54−56^ resulting in five targeted Ir(III) complexes, **DTBP**, **DPPH**, **DOMP**, **FOMP**, and **DFBP**, respectively. The TrPL results show that they all have a microsecond emission, indicating phosphorescent properties, and the PLQYs are greater than 40%. Through application as EML materials for LECs, **DTBP** performs the best with an EQ*E*_max_ value of 10.30%. To achieve a better breakthrough in efficiency, we optimized the thickness of the EML of the **DTBP** LEC device. As the thickness increases, the efficiency shows a positive trend without a noticeable shift in the emission. The best performance was obtained when the thickness of the EML was 507 nm, showing an EQE_max_ of 12.80%. Furthermore, with the introduction of the diffusive substrates, the efficiency of **DTBP** has reached CE_max_ up to 69.37 cd A^–1^, PE_max_ up to 82.87 lm W^1–^, and EQE_max_ up to 22.15%. These are state-of-the-art values among the reported blue-green LECs.

## Supporting Information Available

Crystal data, ^1^H and ^13^C NMR spectra, transient PL curves, TD-DFT calculation, high resolution mass spectra, LECs performance on DTBP with various EML thickness, and synthetic procedure and characterization data of the complexes (PDF)
