# Supplementary material for: Cationic Ir(III) Complexes with 4-Fluoro-4′-pyrazolyl-(1,1′-biphenyl)-2-carbonitrile as the Cyclometalating Ligand: Synthesis, Characterizations, and Application to Ultrahigh-Efficiency Light-Emitting Electrochemical Cells
Source: Inorg Chem. 2024 Mar 6;63(11):4828–38. doi: 10.1021/acs.inorgchem.3c03517 (PMC10951952; doi:10.1021/acs.inorgchem.3c03517)
Supplement: Supplementary file 1 — ic3c03517_si_001.pdf [file ic3c03517_si_001.pdf]

Supporting information

Cationic Ir(III) Complexes with 4-Fluoro-4'-pyrazolyl-(1,1'-biphenyl)-2-carbonitrile as Cyclometalating Ligand: Synthesis, Characterizations, and Application to Ultrahigh-Efficiency Light-Emitting Electrochemical Cells (LECs)

Rong-Huei Yi,[a] Yi-Hsun Lee,[b] Yu-Ting Huang,[a] Xuan-Jun Chen,[b] Yun-Xin Wang,[a] Dian Luo,[b] Chin-Wei Lu,[a,]* and Hai-Ching Su[b,]*

[a] *Department of Applied Chemistry, Providence University, Taichung 43301, Taiwan*

[b] *Institute of Lighting and Energy Photonics, National Yang Ming Chiao Tung University, Tainan 71150, Taiwan*

* Corresponding authors

E-mail: cwlu@pu.edu.tw; Fax: +886-4-26327554; Tel: +886-4-26328001-15213 (C.-W. Lu)

E-mail: haichingsu@nycu.edu.tw; Fax: +886-6-3032535; Tel: +886-6-3032121-57792 (H.-C. Su)

Table of Contents

1. **General information**……3

2. **Materials and Synthesis**……4

3. **Crystal data**……9

4. **^{1}H and ^{13}C NMR spectra**……11

5. **Transient PL curves**……19

6. **Theoretical calculation**……20

7. **Mass spectra**……24

8. **EL spectra and characteristics of the LECs based on DTBP with various EML thicknesses**……27

9. **Reference**……28

General Information

NMR spectra of compounds were collected on a Bruker Ascend 400 MHz spectrometer at room temperature. Photophysical characteristics of complexes in solutions were collected at room temperature using 1.0×10^{-5} M acetonitrile (MeCN) solutions of all complexes on spectrofluorometer Edinburgh FS5, which were carefully purged with nitrogen prior to measurements. Ultraviolet–visible (UV–Vis) absorption spectra were recorded on Perkin Elmer Lambda 14 spectrophotometer. To gain more insight into the electronic structure of Ir-based iTMCs, complete geometrical optimizations were performed using density functional theory (DFT) with B3LYP/LANL2DZ[Ir]6-31G(d)[F,O,N,C,H] basis set within Gaussian 16. Oxidation and reduction potentials of all complexes were determined by cyclic voltammetry (CV) at a scan rate of 100 mV s^{-1} in MeCN solutions (1.0 mM) on ZIVE SP1. A glassy carbon electrode and a platinum wire were used as the working electrode and the counter electrode, respectively. All potentials were recorded versus the Ag/AgCl (sat'd) reference electrode. For reduction voltammograms, 0.1 M tetra-*n*-butylammonium hexafluorophosphate ($TBAPF_6$) in MeCN was used as the supporting electrolyte. Cyclometalated dinuclear iridium complexes with the general formula $(C^\wedge N)_2Ir(\mu\text{-}Cl)_2Ir(C^\wedge N)_2$ were prepared according to literature procedures.[1] Suitable crystal was sealed in thin-walled glass capillaries under a nitrogen atmosphere and mounted on a

Bruker AXS SMART 1000 diffractometer. The absorption correction was based on the symmetry equivalent reflections using the SADABS program. The space group determination was based on a check of the Laue symmetry and systematic absences and was confirmed using the structure solution. The structure was solved by direct methods using a SHELXTL package. All non-H atoms were located from successive Fourier maps, and hydrogen atoms were refined using a riding model. Anisotropic thermal parameters were used for all non-H atoms, and fixed isotropic parameters were used for H atoms.

Materials and Synthesis

Synthesis of 4-fluoro-4'-(1*H*-pyrazol-1-yl)-[1,1'-biphenyl]-2-carbonitrile (ppfn). 1-(4-(4,4,5,5-tetramethyl-1,3,2-dioxaborolan-2-yl)phenyl)-1*H*-pyrazole (0.50 g, 1.9 mmol), 2-bromo-5-fluorobenzonitrile (0.41 g, 2.0 mmol), potassium carbonate (0.77 g, 5.6 mmol) and [1,1'-bis(diphenylphosphino)ferrocene]dichloropalladium(II) ($Pd(dppf)Cl_2$) (0.50 mol %) was dissolved in toluene (5.0 mL), ethanol (0.50 mL) and H_2O (0.50 mL) in a round-bottom flask. The reaction mixture was refluxed for 24 h. After being cooled to room temperature, the mixture was extracted with ethyl acetate. The organic phase was subsequently washed with water and then dried over anhydrous sodium sulfate (Na_2SO_4). The solvent was evaporated under vacuum and the residual

was purified through column chromatography on silica gel using ethyl acetate/*n*-hexane (1/6, v/v) as the eluent, affording a white solid (0.42 g, 88%). ^{1}H NMR (400 MHz, $CDCl_3$) δ 7.99 (d, J = 2.5 Hz, 1H), 7.84 (d, J = 8.6 Hz, 2H), 7.76 (s, 1H), 7.62 (d, J = 8.6 Hz, 2H), 7.54–7.51 (m, 1H), 7.48 (dd, J = 8.1, 2.7 Hz, 1H), 7.39 (td, J = 8.3, 2.7 Hz, 1H), 6.51 (s, 1H).

Synthesis of 5-fluoro-5'-methoxy-2,2'-bipyridine (fomp). Sodium hydride (0.11 g, 3.0 mmol) was dissolved in dimethylformamide (8.0 mL) in a round-bottom flask placed in ice bath. Subsequently, 5,5'-difluoro-2,2'-bipyridine (0.30 g, 1.6 mmol) was dissolved in methanol (0.50 mL) and added to the flask. The reaction continued for 17 hours. After the reaction was completed, the mixture was subjected to extraction with ethyl acetate/H_2O and then dried using anhydrous Na_2SO_4. The solvent was removed under vacuum and the residual was purified through column chromatography on silica gel using *n*-hexane as the eluent, affording **fomp** (0.21 g, 51%) as a white solid. ^{1}H NMR (400 MHz, $CDCl_3$) δ 8.47 (d, J = 2.8 Hz, 1H), 8.35–8.30 (m, 2H), 8.28 (d, J = 8.8 Hz, 1H), 7.49 (td, J = 8.5, 2.9 Hz, 1H), 7.31 (dd, J = 8.8, 3.0 Hz, 1H), 3.92 (s, 3H). ^{13}C NMR (101 MHz, DMSO-d_6) δ 160.37 (s), 157.84 (s), 155.93 (s), 151.96 (d, J = 3.1 Hz), 147.06 (s), 136.99 (t, J = 10.1 Hz), 124.14 (d, J = 18.2 Hz), 121.29 (d, J = 4.2 Hz), 121.22 (s), 121.15 (s), 55.73 (s).

Synthesis of [Ir_2(ppfn)$_4Cl_2$]. ppfn (0.88 g, 3.4 mmol) was dissolved in 2-

ethoxyethanol (40 mL) in a round-bottom flask. Subsequently, Ir(III) chloride hydrate (0.50 g, 1.7 mmol) and 10 mL of water were added to the flask. The reaction mixture was refluxed for 24 h and then cooled to room temperature. The resulting precipitate was collected, washed with water, and dried under vacuum to obtain chloro-bridged Ir dimer **[Ir_2(ppfn)$_4$$Cl_2$]** (1.1 g, 89%) as a gray solid, which was used directly without the need for further purification.

Synthesis of di[4-fluoro-4'-pyrazolyl-(1,1'-biphenyl)-2-carbonitrile]-4,4'-di-*tert*-butyl-2,2'-bipyridyl iridium(III) hexafluorophosphate (DTBP). In a round-bottomed flask, **[Ir_2(ppfn)$_4$$Cl_2$]** (0.50 g, 0.33 mmol) and 4,4'-di-*tert*-butyl-2,2'-bipyridine (**dtbp**) (0.18 mg, 0.66 mmol) were combined in 40 mL of methanol. The solution was then refluxed for 24 h under an inert atmosphere. After cooling to room temperature, a counterion exchange from Cl^- to PF_6^- was performed through a metathesis reaction. Complexes were precipitated from the methanol solution by adding an excess of KPF_6, followed by washing with water and *n*-hexane, and drying under vacuum. The crude product was further purified by column chromatography on silica gel using dichloromethane as the eluent to give **DTBP** (0.52 g, 80%) as a yellow solid. ^{1}H NMR (400 MHz, $CDCl_3$) δ 8.27 (s, 2H), 8.24 (d, J = 5.9 Hz, 2H), 8.13 (s, 2H), 7.53–7.47 (m, 4H), 7.38–7.31 (m, 6H), 7.16 (d, J = 8.2 Hz, 2H), 7.02 (s, 2H), 6.58 (t, J = 2.6 Hz, 2H), 6.54 (s, 2H), 1.45 (s, 18H). ^{13}C NMR (101 MHz, CD_3CN) δ 165.3, 163.3,

160.8, 157.2, 151.9, 144.8, 142.7, 139.8, 136.1, 134.3, 133.8, 133.0, 129.7, 125.8, 124.9, 122.6, 121.4, 120.9, 113.3, 112.8, 109.2, 36.4, 30.4. HRMS (ESI$^+$) m/z: calcd for $C_{50}H_{42}N_8F_2Ir^+$ [M]$^+$: 985.3124, found: 985.3107. Anal. calcd for $C_{50}H_{42}F_8IrN_8P$: C, 53.14; H, 3.75; N, 9.92, found C, 53.64; H, 3.50; N, 10.31.

Synthesis of di[4-fluoro-4'-pyrazolyl-(1,1'-biphenyl)-2-carbonitrile]-4,7-diphenyl-1,10-phenanthroline iridium(III) hexafluorophosphate (DPPH). The synthesis of **DPPH** followed a procedure similar to that for **DTBP**, with 4,7-diphenyl-1,10-phenanthroline (**dpph)** being used instead of **dtbp**, resulting in a yellow solid (0.39 g, 62%). ^{1}H NMR (400 MHz, $CDCl_3$) δ 8.71 (d, J = 5.2 Hz, 2H), 8.18–8.13 (m, 4H), 7.81 (d, J = 5.0 Hz, 2H), 7.63–7.54 (m, 12H), 7.45 (d, J = 8.2 Hz, 2H), 7.38 (d, J = 8.1 Hz, 4H), 7.23 (d, J = 8.2 Hz, 2H), 7.08 (s, 2H), 6.71 (s, 2H), 6.56 (s, 2H). ^{13}C NMR (101 MHz, CD_3CN) δ 163.2, 160.8, 152.3, 151.9, 148.9, 145.0, 142.7, 140.2, 136.6, 136.1, 134.4, 133.2, 132.9, 130.63, 130.59, 130.2, 130.0, 129.7, 127.3, 126.9, 125.0, 121.4, 120.9, 113.3, 112.8, 109.1. HRMS (ESI$^+$) m/z: calcd for $C_{56}H_{34}N_8F_2Ir^+$ [M]$^+$: 1049.2498, found: 1049.2474. Anal. calcd for $C_{56}H_{34}F_8IrN_8P$: C, 56.33; H, 2.87; N, 9.38, found C, 56.63; H, 3.14; N, 9.58.

Synthesis of di[4-fluoro-4'-pyrazolyl-(1,1'-biphenyl)-2-carbonitrile]-5,5'-dimethoxyl-2,2'-dipyridyl iridium(III) hexafluorophosphate (DOMP). The synthesis of **DOMP** followed a procedure similar to that for **DTBP**, with 5,5'-

dimethoxy-2,2'-bipyridine (**domp**) being used instead of **dtbp** , resulting in a yellow solid (0.51 g, 82%).^{1}H NMR (400 MHz, DMSO-d_6) δ 8.90 (s, 2H), 8.69 (d, J = 9.2 Hz, 2H), 7.90–7.81 (m, 6H), 7.59–7.64 (m, 6H), 7.26–7.28 (m, 4H), 6.74 (t, J = 2.6 Hz, 2H), 6.53 (d, J = 1.9 Hz, 2H), 3.76 (s, 6H). ^{13}C NMR (101 MHz, CD_3CN) δ 163.2, 160.8, 158.8, 149.8, 144.9, 142.5, 140.2, 139.5, 135.9, 134.3, 133.4, 132.9, 129.8, 125.2, 125.0, 124.6 (d, J = 24.5 Hz), 121.4, 120.9, 113.3, 112.7, 109.1, 57.0. HRMS (ESI$^+$) m/z: calcd for $C_{44}H_{30}N_8O_2F_2Ir^+$ [M]$^+$: 933.2084, found: 933.2059. Anal. calcd for $C_{44}H_{30}O_2F_8IrN_8P$: C, 49.03; H, 2.81; N, 10.40, found C, 48.88; H, 2.62; N, 10.38.

Synthesis of di[4-fluoro-4'-pyrazolyl-(1,1'-biphenyl)-2-carbonitrile]-5-fluoro-5'-methoxyl-2,2'-bipyridyl iridium(III) hexafluorophosphate (FOMP). The synthesis of **FOMP** followed a procedure similar to that for **DTBP**, with **fomp** being used instead of **dtbp**, resulting in a yellow solid (0.46 g, 75%). ^{1}H NMR (400 MHz, DMSO-d_6) δ 8.90–8.88 (m, 2H), 8.86–8.82 (m, 1H), 8.78 (d, J = 9.2 Hz, 1H), 8.27 (t, J = 8.4 Hz,1H), 7.98–7.79 (m, 6H), 7.68 (s, 1H), 7.64–7.54 (m, 4H), 7.36 (s, 1H), 7.32–7.23 (m, 3H), 6.75 (t, J = 2.5 Hz, 2H), 6.54 (s, 1H), 6.48 (s, 1H), 3.79 (s, 3H). ^{13}C NMR (101 MHz, DMSO-d_6) δ 162.0, 159.5, 158.8, 158.2, 153.1, 147.5, 143.6, 141.2, 139.7, 138.7, 134.4, 132.6, 132.1, 131.6, 129.7, 127.2, 126.1, 125.6, 124.3, 123.6, 121.0, 120.3, 117.6, 112.8, 111.1, 108.7, 56.3. HRMS (ESI$^+$) m/z: calcd for $C_{43}H_{27}N_8OF_3Ir^+$ [M]$^+$: 921.1884, found: 921.1870. Anal. calcd for $C_{43}H_{27}OF_9IrN_8P$: C, 48.45; H, 2.55; N,

10.51, found C, 48.31; H, 2.22; N, 10.38.

Synthesis of di[4-fluoro-4'-pyrazolyl-(1,1'-biphenyl)-2-carbonitrile]-5,5'-difluoro-2,2'-bipyridine iridium(III) hexafluorophosphate (DFBP). The synthesis of **DFBP** followed a procedure similar to that for **DTBP**, with 5,5'-difluoro-2,2'-bipyridine (**dfbp**) being used instead of **dtbp**, resulting in a yellow solid (0.49 g, 74%). ^{1}H NMR (400 MHz, CD_3CN) δ 8.31 (m, 2H), 8.17 (d, J = 2.9 Hz, 2H), 7.87 (m, 2H), 7.77 (t, J = 9.6 Hz, 2H), 7.33 (m, 6H), 7.21 (t, J = 9.8 Hz, 2H), 7.03 (d, J = 8.2 Hz, 2H), 6.94 (s, 2H), 6.39 (t, J = 2.6 Hz, 2H), 6.34 (s, 2H). ^{13}C NMR (101 MHz, CD_3CN) δ 163.3, 160.7, 153.2, 144.8, 142.6, 141.6, 141.3, 140.4, 136.1, 134.2, 132.9, 131.8, 130.0, 127.7, 127.2, 125.4, 121.4, 120.9, 118.3, 113.5, 112.9, 109.2. HRMS (ESI$^+$) m/z: calcd for $C_{42}H_{24}N_8F_4Ir^+$ [M]$^+$: 909.1684, found: 909.1670. Anal. calcd for $C_{42}H_{24}F_{10}IrN_8P$: C, 47.87; H, 2.30; N, 10.63, found C, 47.58; H, 2.59; N, 11.08.

Table S1. Crystal data of complex **DTBP**.

Complex	**DTBP (CCDC 2168344)**
Empirical formula	$C_{50}H_{42}F_8Ir_8N_8P$
Formula weight	1130.12
Temperature	150(2) K
Wavelength	0.71073 Å
Crystal system	Triclinic
Space group	P-1

Unit cell dimensions	a = 12.5675(4) Å α= 94.8890(10)°.<br>b = 12.6404(4) Å β= 103.1040(10)°.<br>c = 18.8126(6) Å γ = 103.5740(10)°.
Volume	2799.00(15) Å ^{3}
Z	2
Density (calculated)	1.624 Mg/m^3
Absorption coefficient	2.770 mm^{-1}
F(000)	1356
Crystal size	0.260 x 0.100 x 0.050 mm^3
Theta range for data collection	2.459 to 27.906°.
Index ranges	-16<=h<=14, -16<=k<=16, -24<=l<=24
Reflections collected	58796
Independent reflections	13365 [R(int) = 0.0527]
Completeness to theta = 25.242°	99.8 %
Absorption correction	Semi-empirical from equivalents
Max. and min. transmission	0.7456 and 0.6192
Refinement method	Full-matrix least-squares on F^2
Data / restraints /	13365 / 3 / 673

parameters	
Goodness-of-fit on F^2	1.036
Final R indices [I>2sigma(I)]	R1 = 0.0373, wR2 = 0.0908
R indices (all data)	R1 = 0.0453, wR2 = 0.0953
Extinction coefficient	n/a
Largest diff. peak and hole	2.375 and -1.780 e.Å $^{-3}$

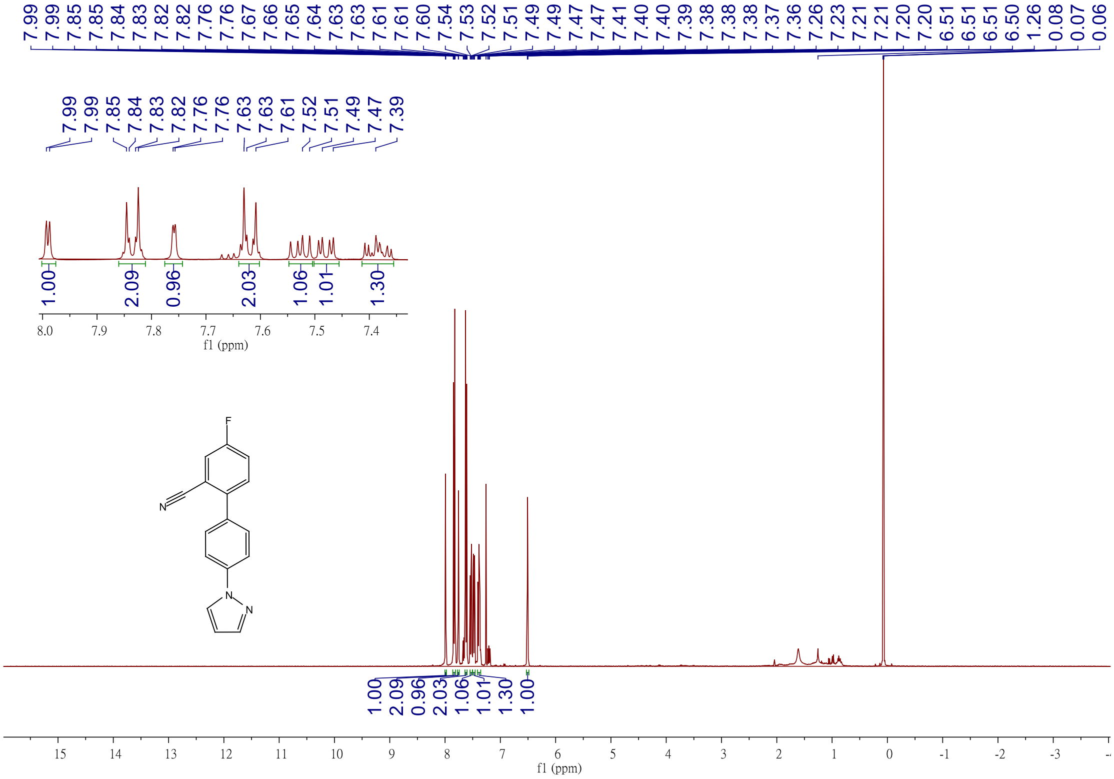

Figure S1. ^{1}H NMR spectrum for **ppfn** (400 MHz, $CDCl_3$, 298 K).

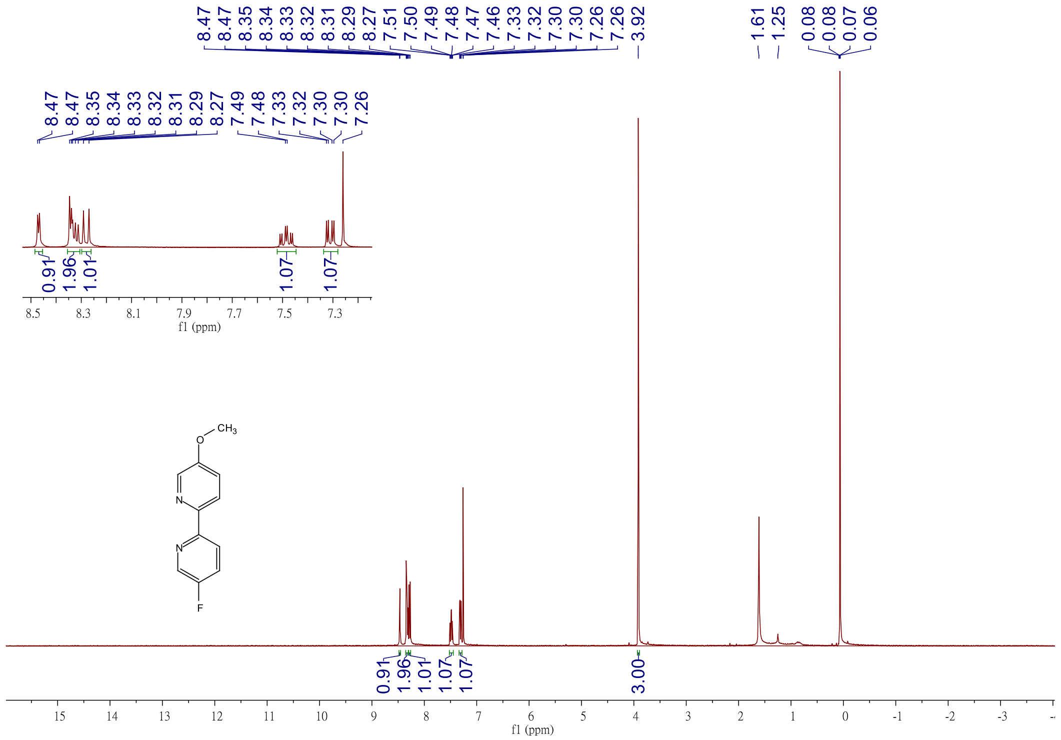

Figure S2. ^{1}H NMR spectrum for **fomp** (400 MHz, $CDCl_3$, 298 K)

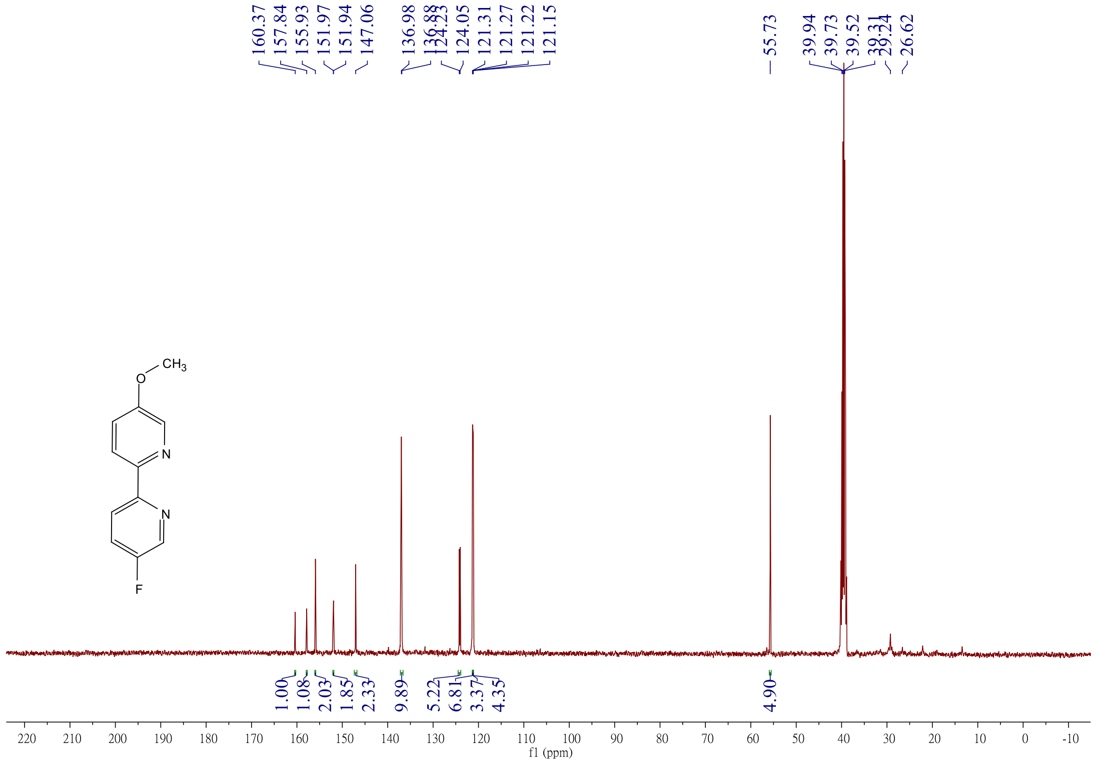

Figure S3. ^{13}CNMR spectrum for **fomp** (101 MHz, DMSO-d_6, 298 K)

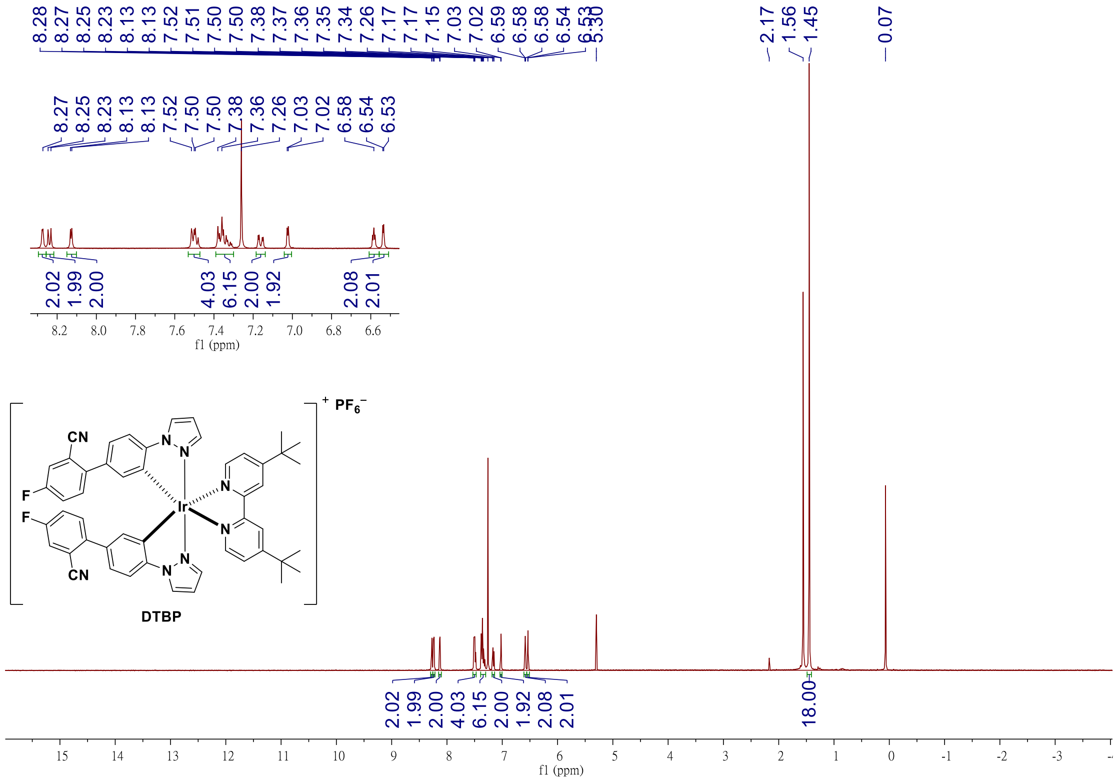

Figure S4. ^{1}H NMR spectrum for **DTBP** (400 MHz, $CDCl_3$, 298 K)

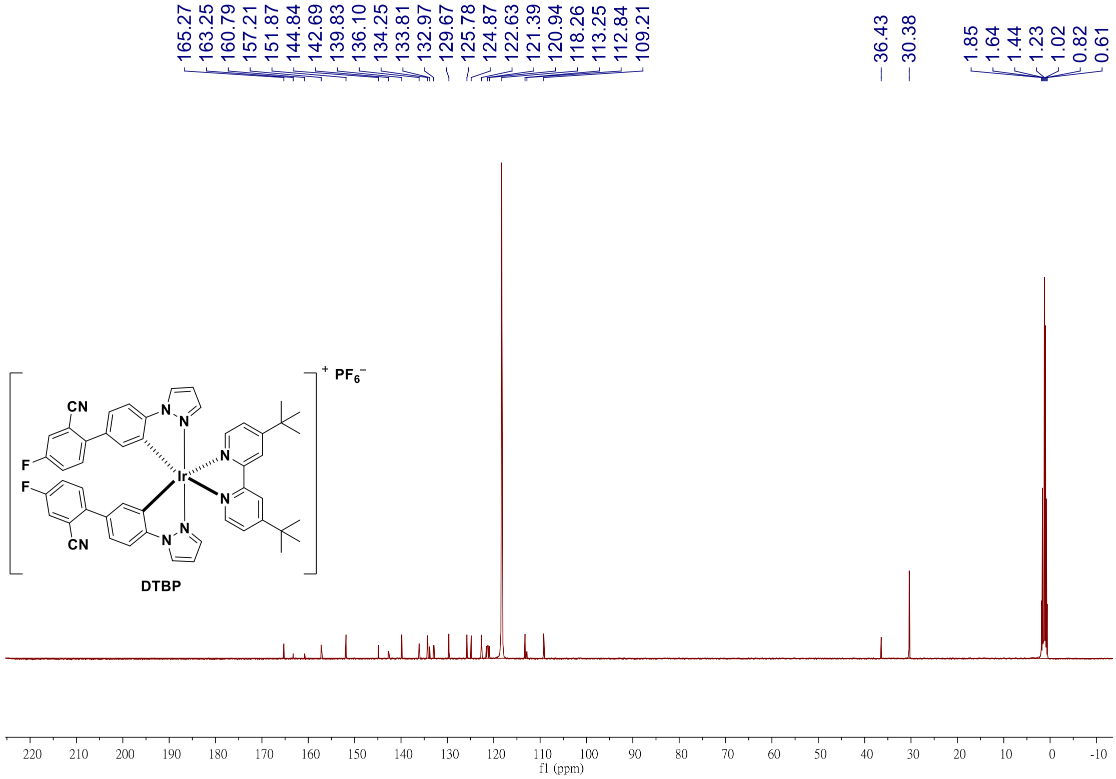

Figure S5. ^{13}C NMR spectrum for **DTBP** (101 MHz, CD_3CN, 298 K)

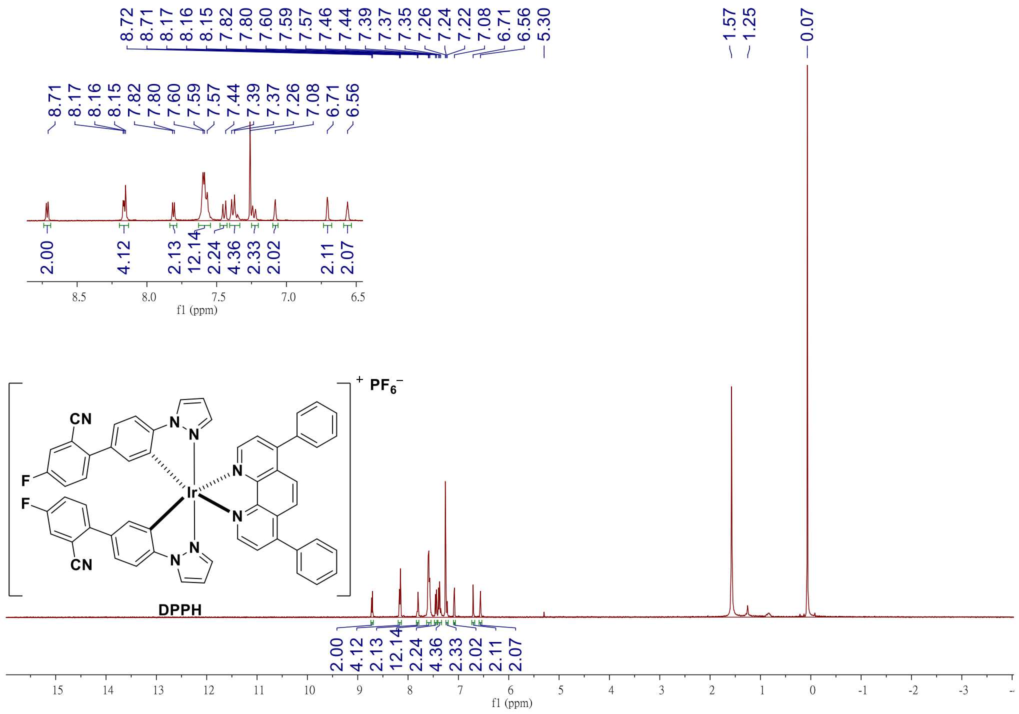

Figure S6. ^{1}H NMR spectrum for **DPPH** (400 MHz, $CDCl_3$, 298 K)

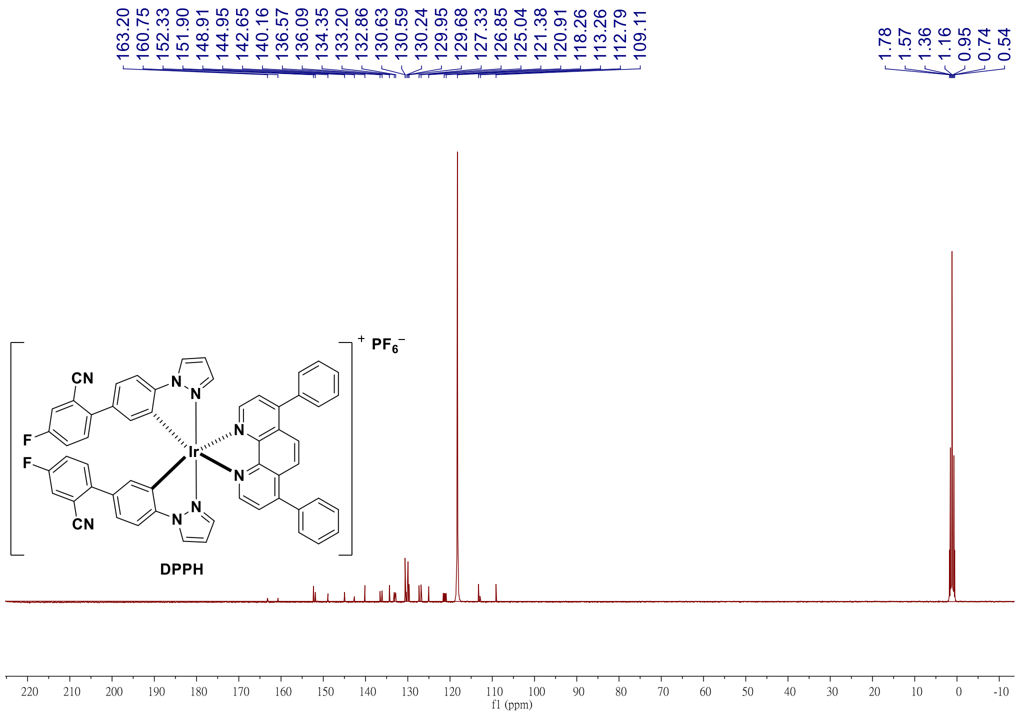

Figure S7. ^{13}C NMR spectrum for **DPPH** (101 MHz, CD_3CN, 298 K)

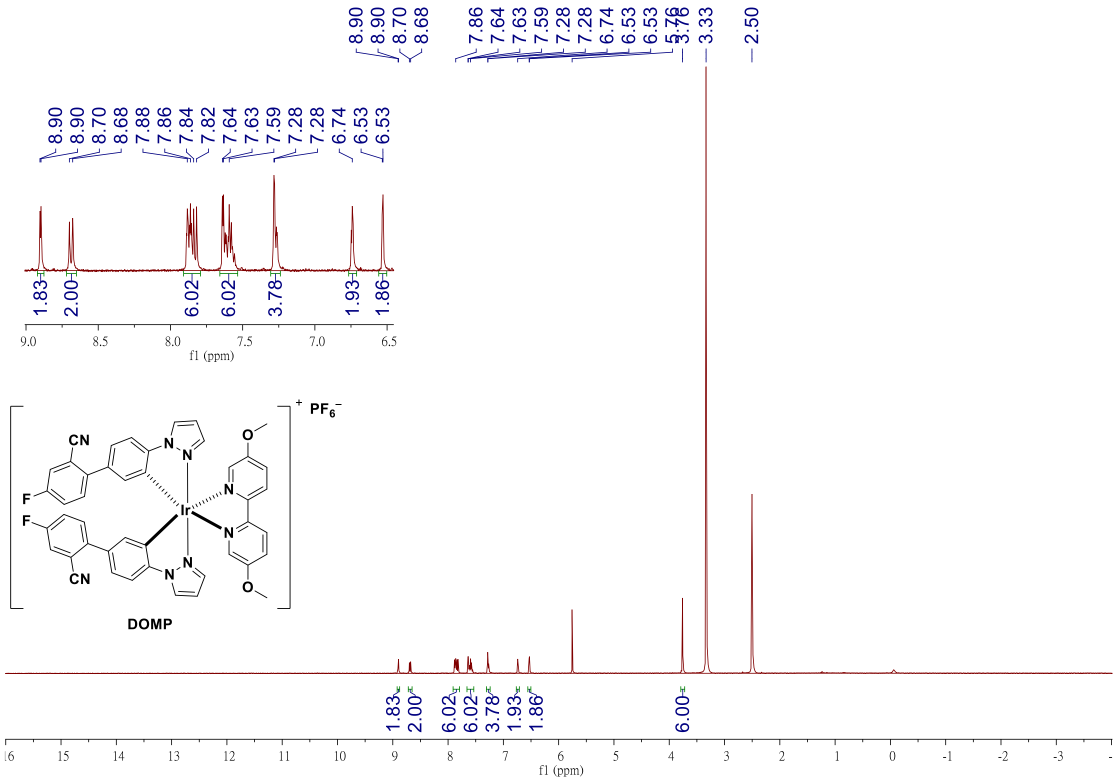

Figure S8. ^{1}H NMR spectrum for **DOMP** (400 MHz, DMSO-d_6, 298 K)

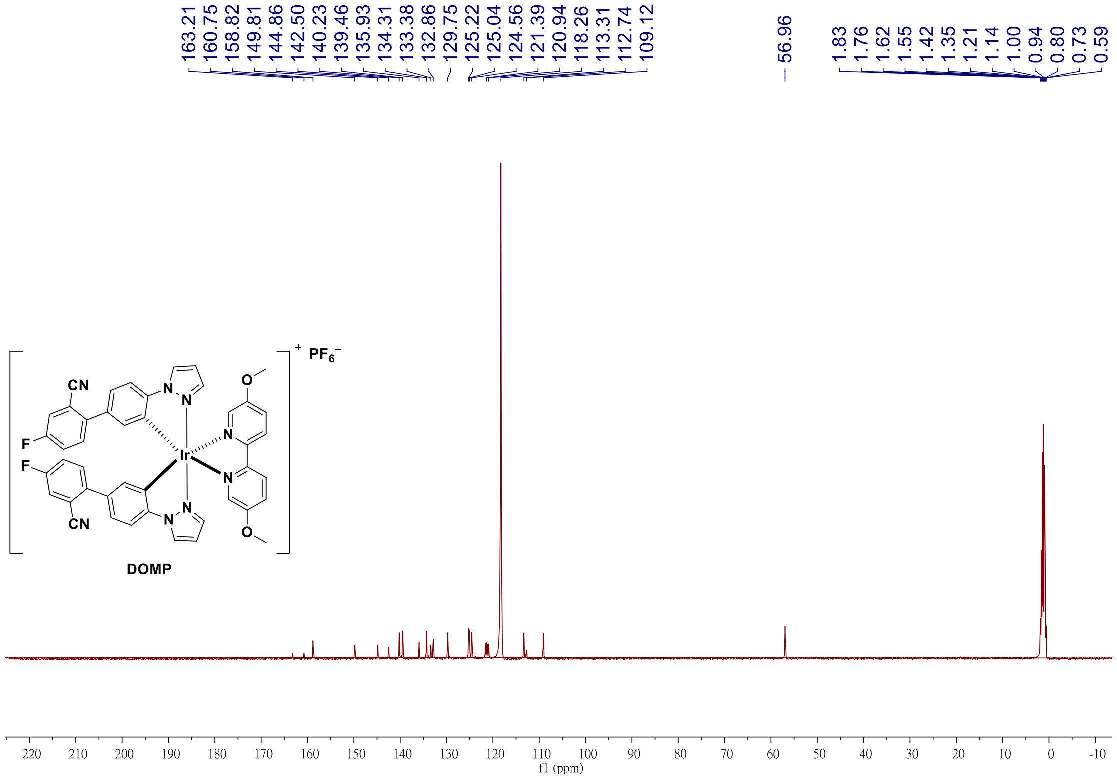

Figure S9. ^{13}C NMR spectrum for **DOMP** (101 MHz, CD_3CN, 298 K)

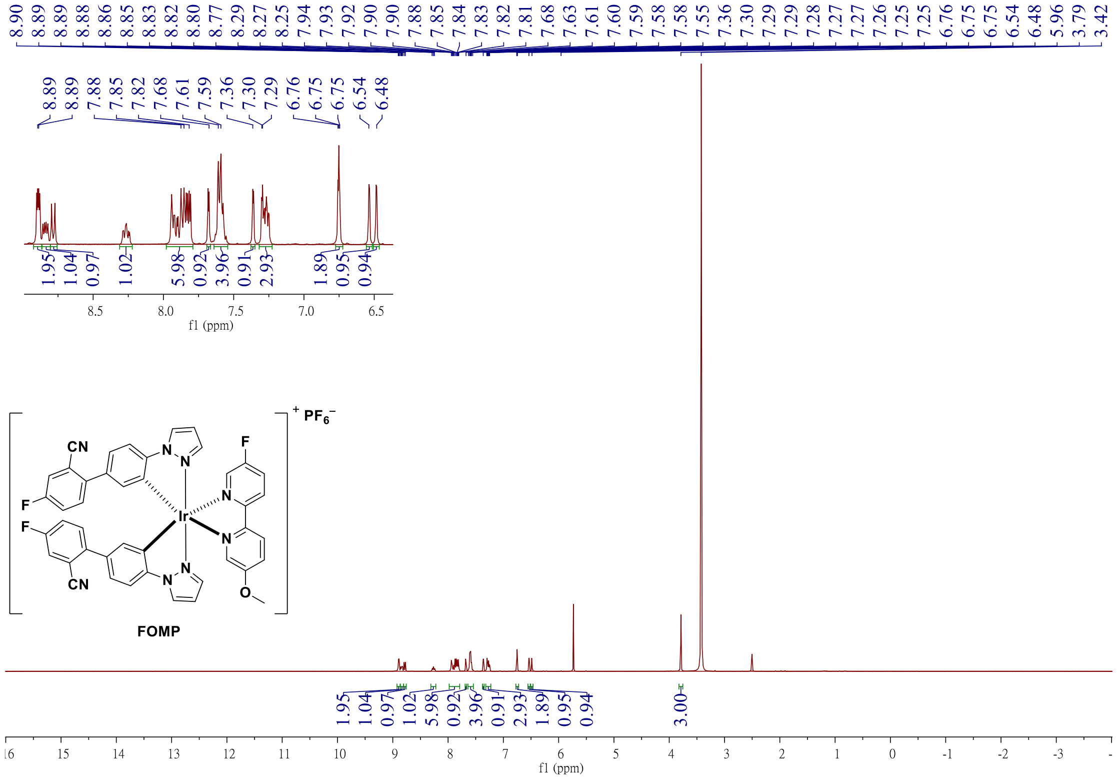

Figure S10. ^{1}H NMR spectrum for **FOMP** (400 MHz, DMSO-d_6, 298 K)

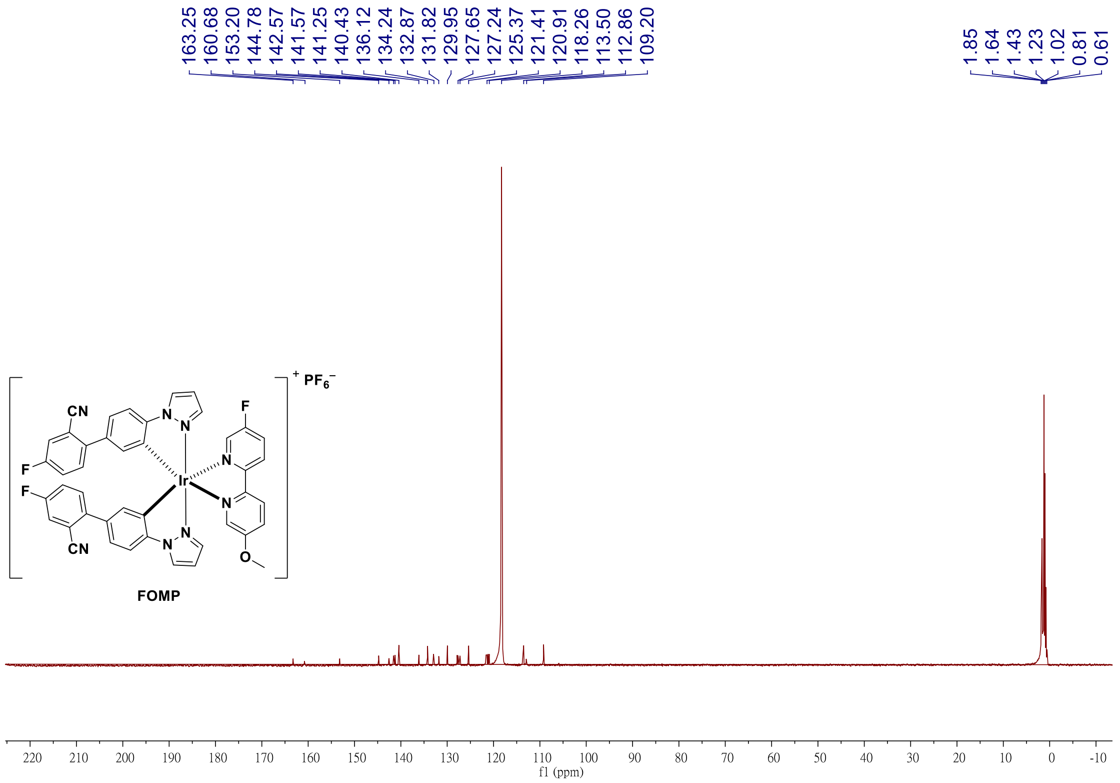

Figure S11. ^{13}C NMR spectrum for **FOMP** (101 MHz, DMSO-d_6, 298 K)

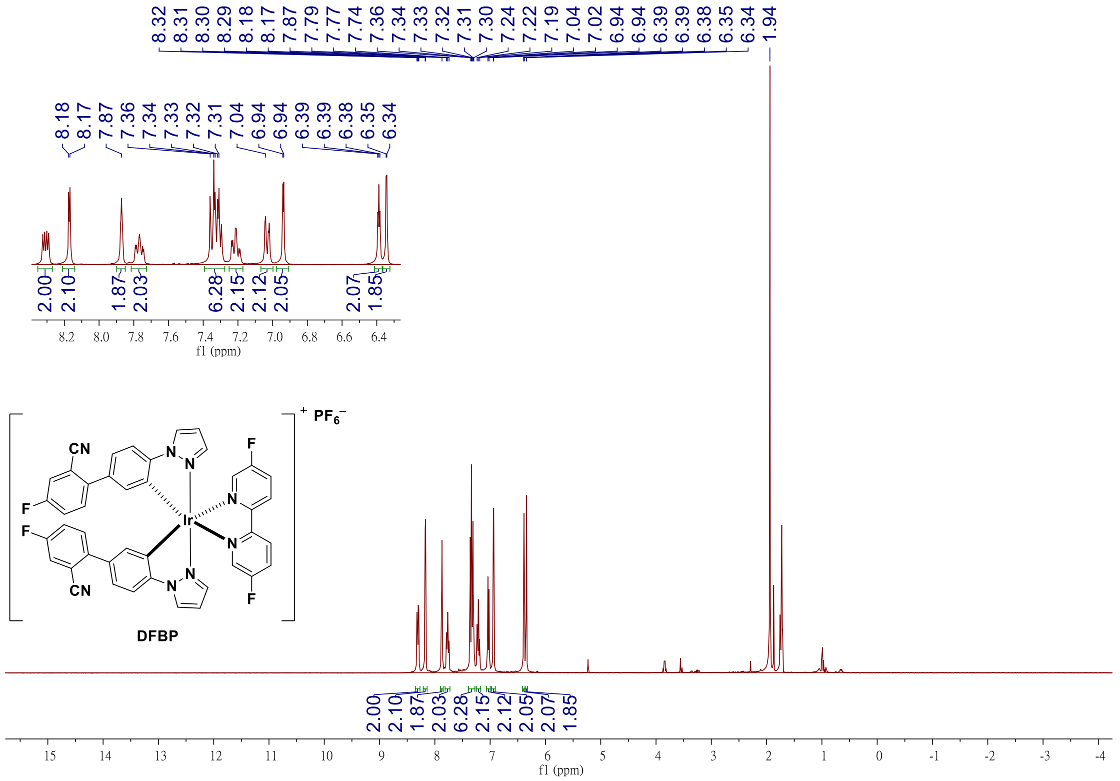

Figure S12. ^{1}H NMR spectrum for **DFBP** (400 MHz, CD_3CN, 298 K)

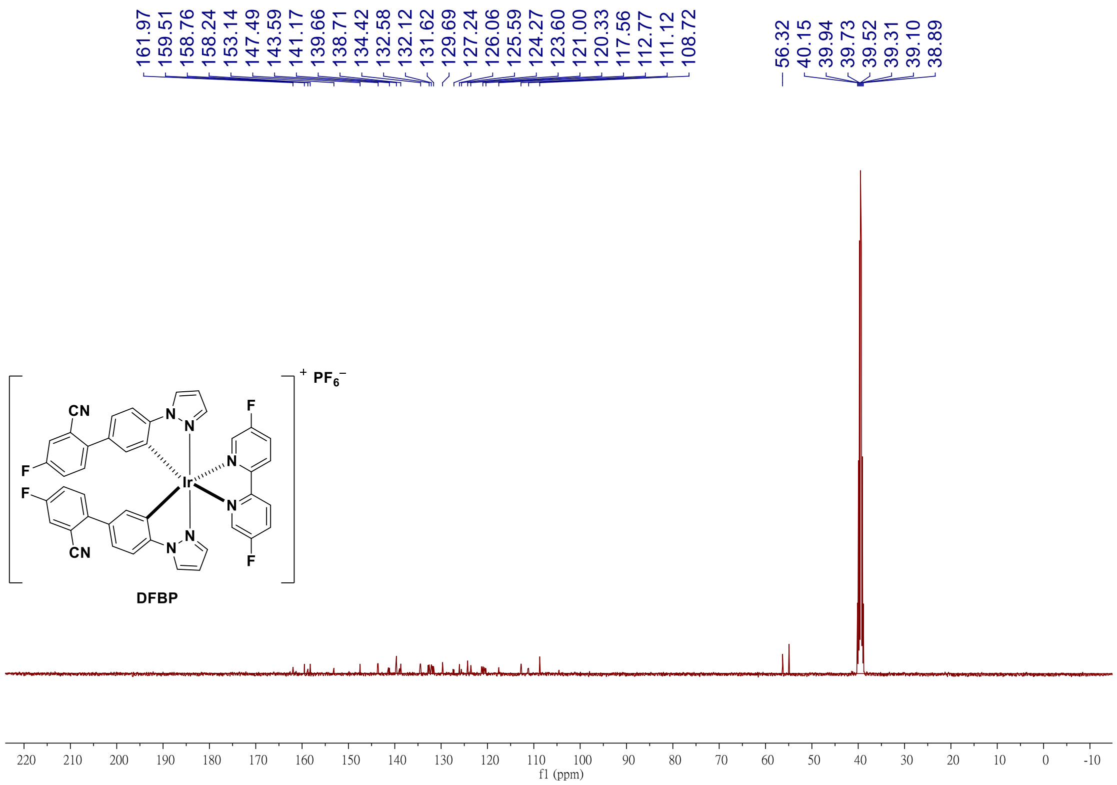

Figure S13. ^{13}C NMR spectrum for **DFBP** (101 MHz, CD_3CN, 298 K)

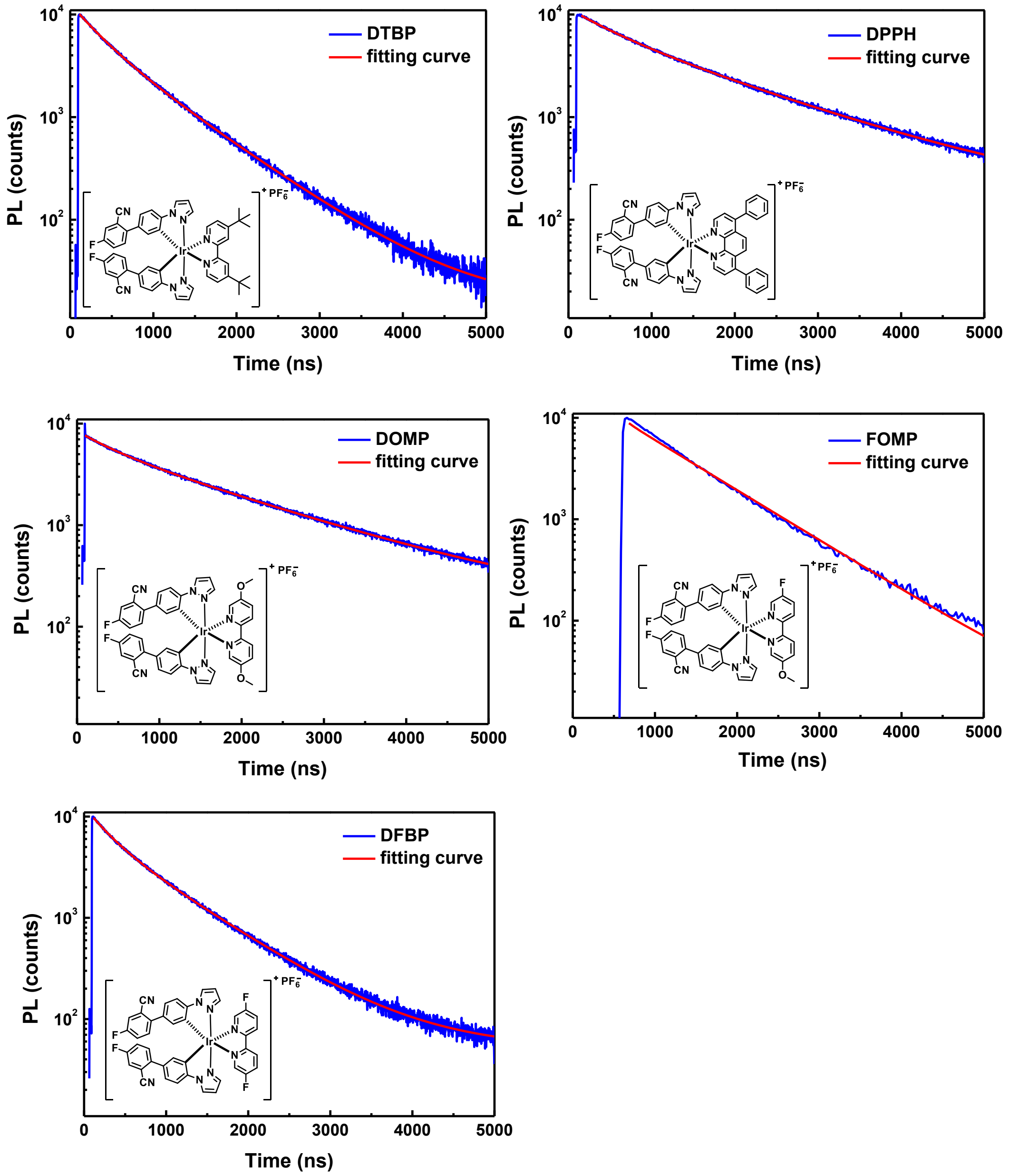

Figure S14. Transient photoluminescence decay and fitting curve of complex **DTBP**–**DFBP** in the thin-film contained complex (80 wt.%) and [BMIM$^+$(PF$_6$)$^-$] (20 wt.%) at 298 K after excitation at 365 nm.

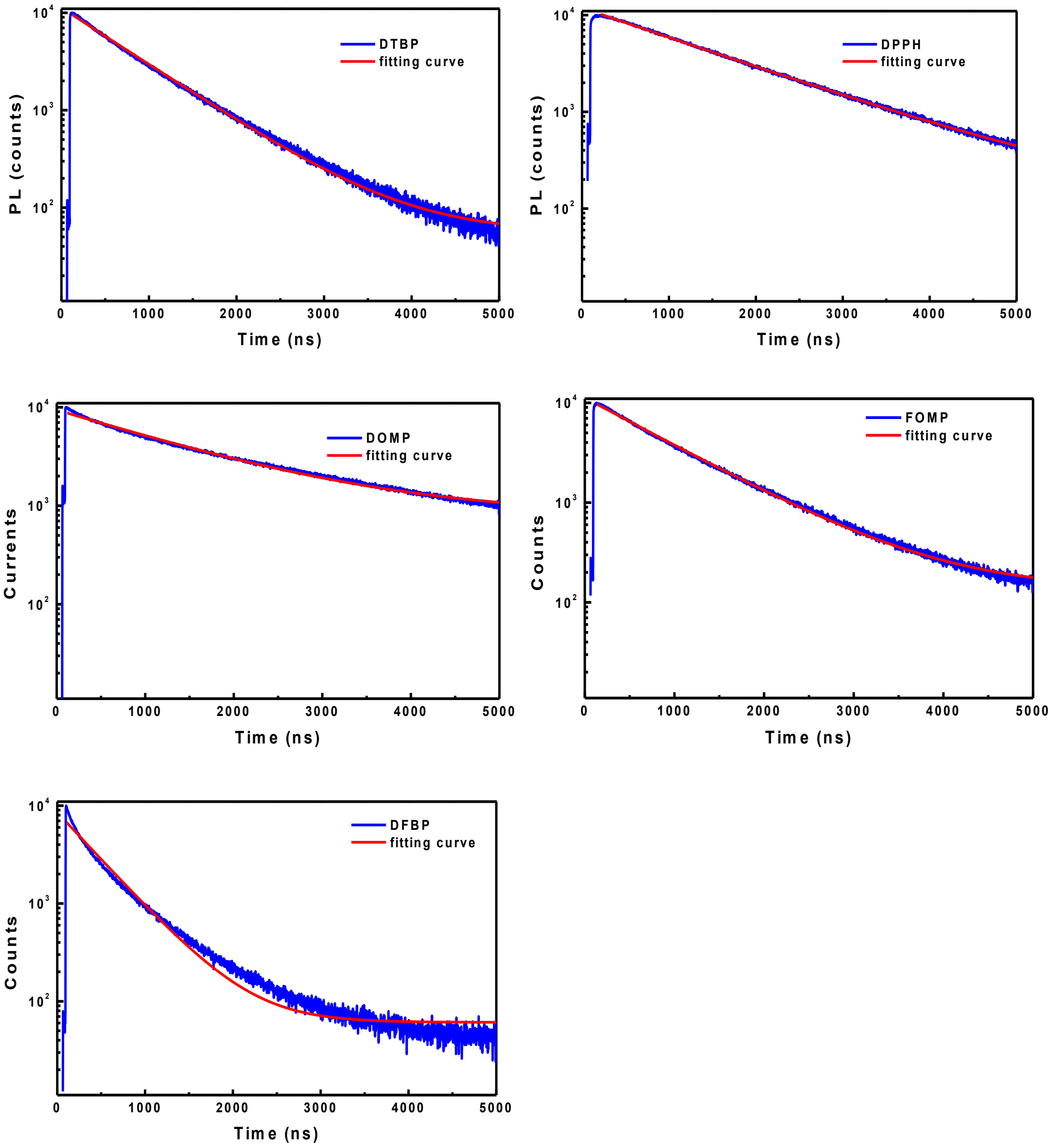

Figure S15. Transient photoluminescence decay and fitting curve of complex **DTBP–DFBP** in neat film at 298 K after excitation at 365 nm.

Table S2. Calculated triplet excited states for the cationic iridium complex **DTBP–DFBP** by TD-DFT approach.

Complex	States	eV [a)]	Dominant excitations [b)]

**DTBP**	T_1	2.37	H → L (78%)<br>H-2 → L (4%)<br>H → L+1 (4%)<br>H → L+3 (4%)
**DPPH**	T_1	1.59	H → L (40%)<br>H-2 → L (40%)<br>H-5 → L (7%)<br>H-2 ← L (4%)
**DOMP**	T_1	2.16	H → L (92%)<br>H → L+2 (3%)<br>H ← L (2%)
**FOMP**	T_1	2.24	H → L (47%)<br>H-2 → L (25%)<br>H-1 → L (22%)
**DFBP**	T_1	2.41	H-2 → L (52%)<br>H-5 → L (5%)<br>H-4 → L (19%)<br>H-3 → L (14%)<br>H-2 → L (2%)<br>H → L (4%)

[a] Calculated excitation energies for the triplet states in MeCN solution; [b] H and L denote the HOMO and LUMO, respectively; data in parentheses are the contributions of the excitation.

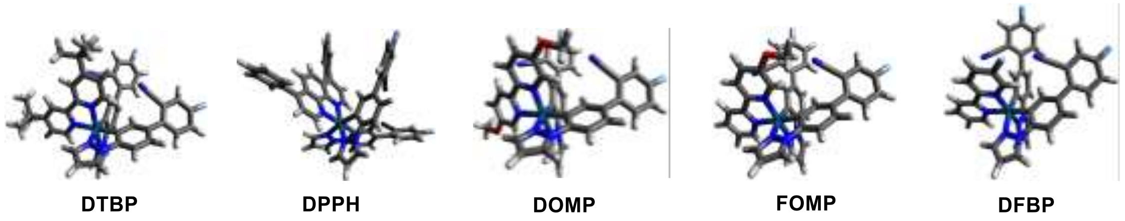

Figure S16. Ground state geometry of complex **DTBP–DFBP** obtain at B3LYP/6-31G(d)/LanL2DZ level of theory.

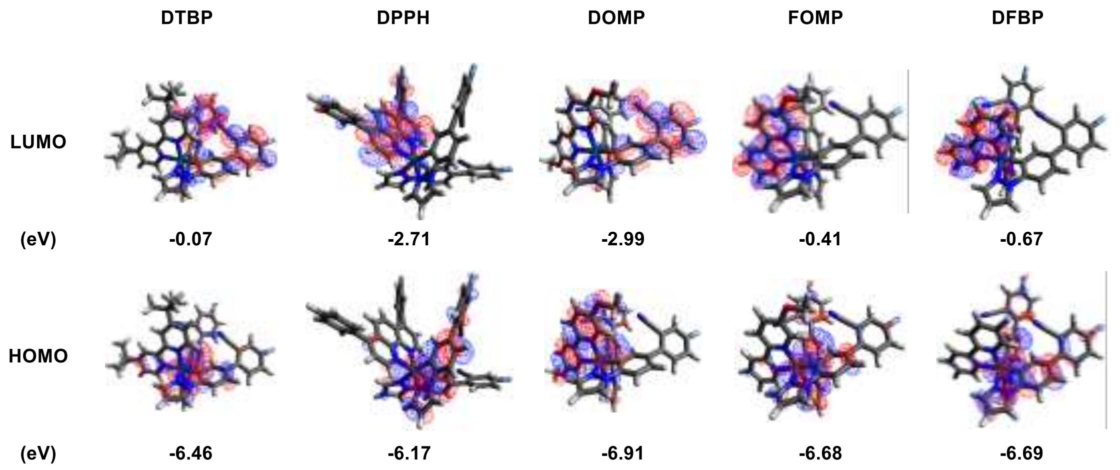

Figure S17. DFT simulations in MeCN of B3LYP/LANL2DZ (**DTBP**, **DOMP**, **FOMP**, and **DFBP**) and B3LYP/DEF2TZVP (**DPPH**) optimized configurations and the LUMO/HOMO orbital distributions in ground state.

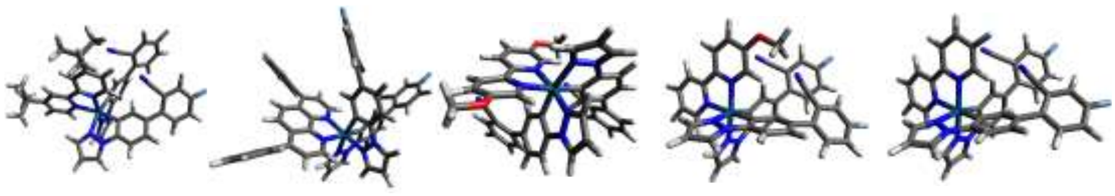

DTBP **DPPH** **DOMP** **FOMP** **DFBP**

Figure S18. T_1 excited state geometry of complex **DTBP–DFBP** obtained in MeCN of m026-2x/LANL2DZ level of theory.

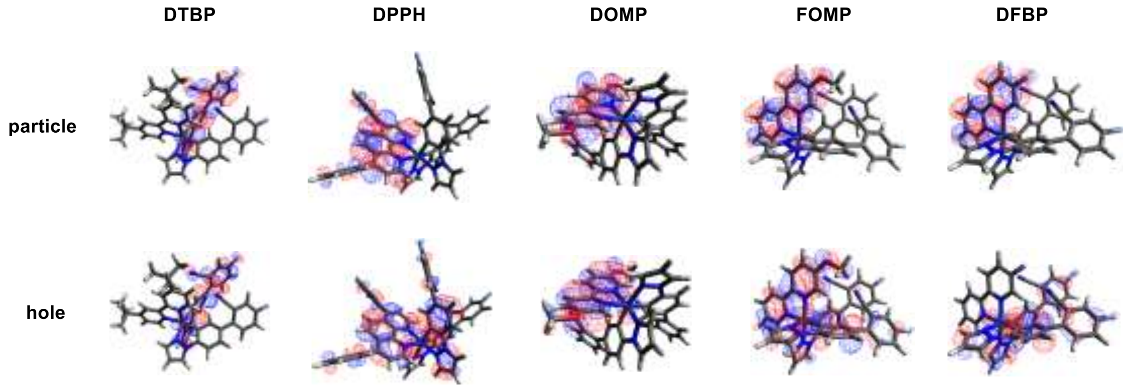

Figure S19. TD-DFT simulations in MeCN of m026-2x/LANL2DZ of T_1 excited state structures and the natural transition orbital (NTO) describing T_1 and S_0 transition between the excited particle and empty hole.

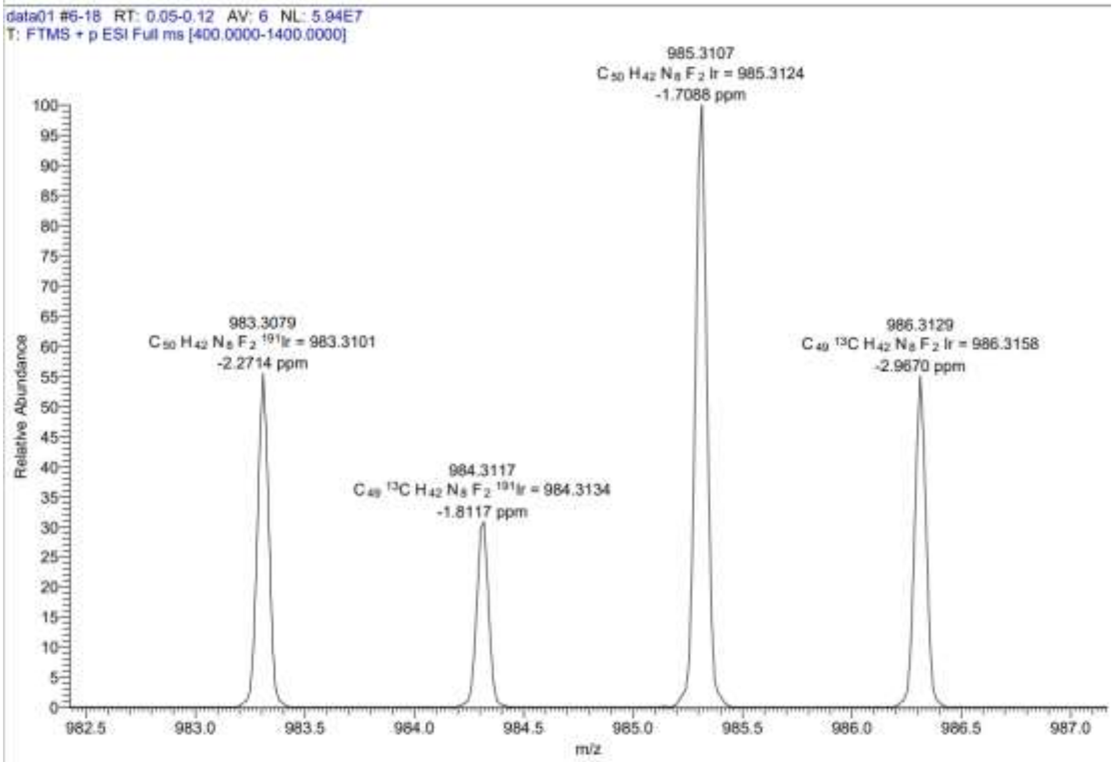

Figure S20. HRMS-ESI$^+$ spectrum of **DTBP**.

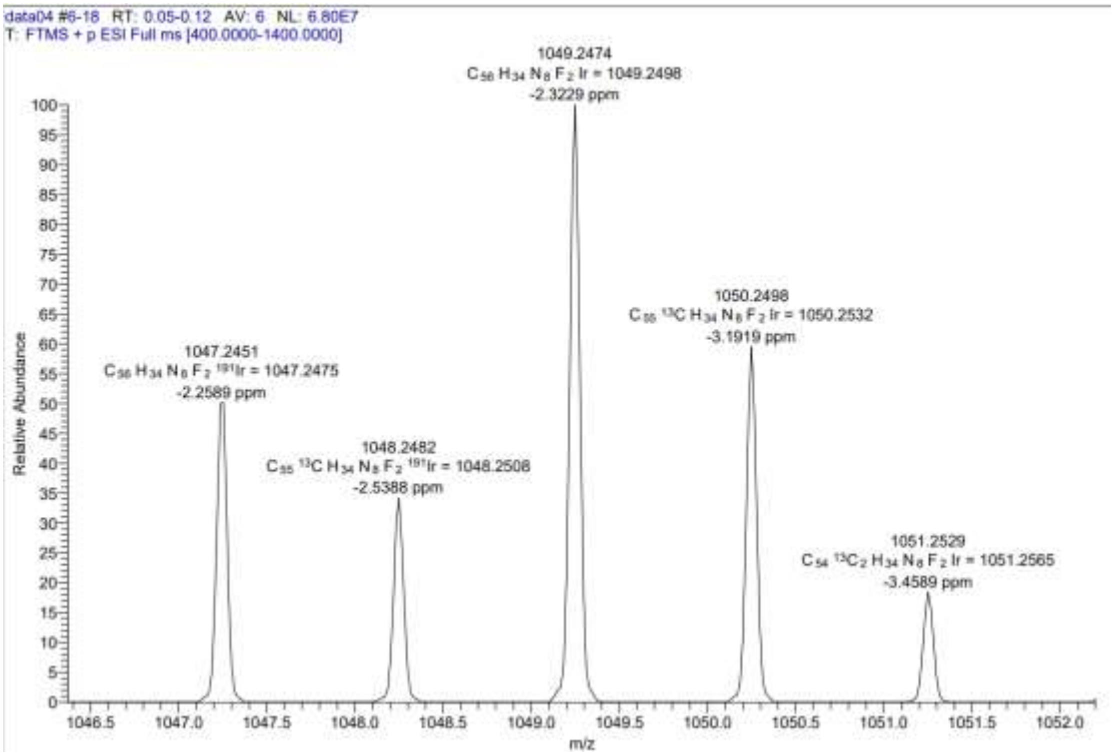

Figure S21. HRMS-ESI$^+$ spectrum of **DPPH**.

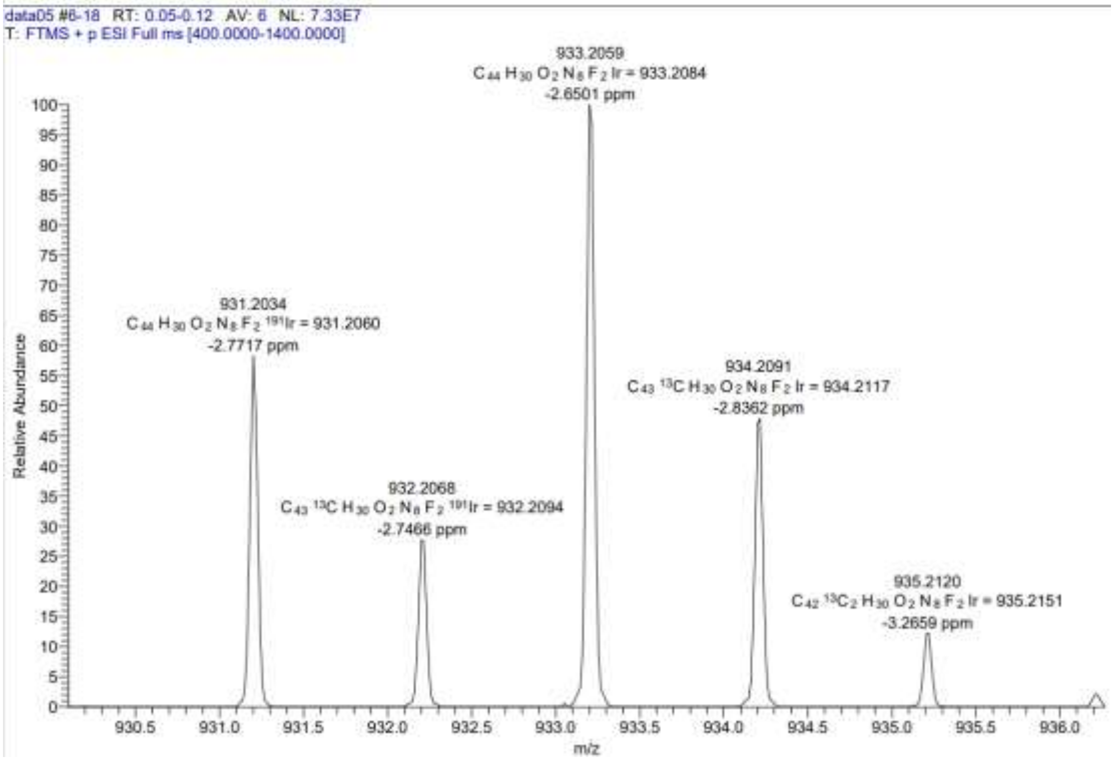

Figure S22. HRMS-ESI$^+$ spectrum of **DOMP**.

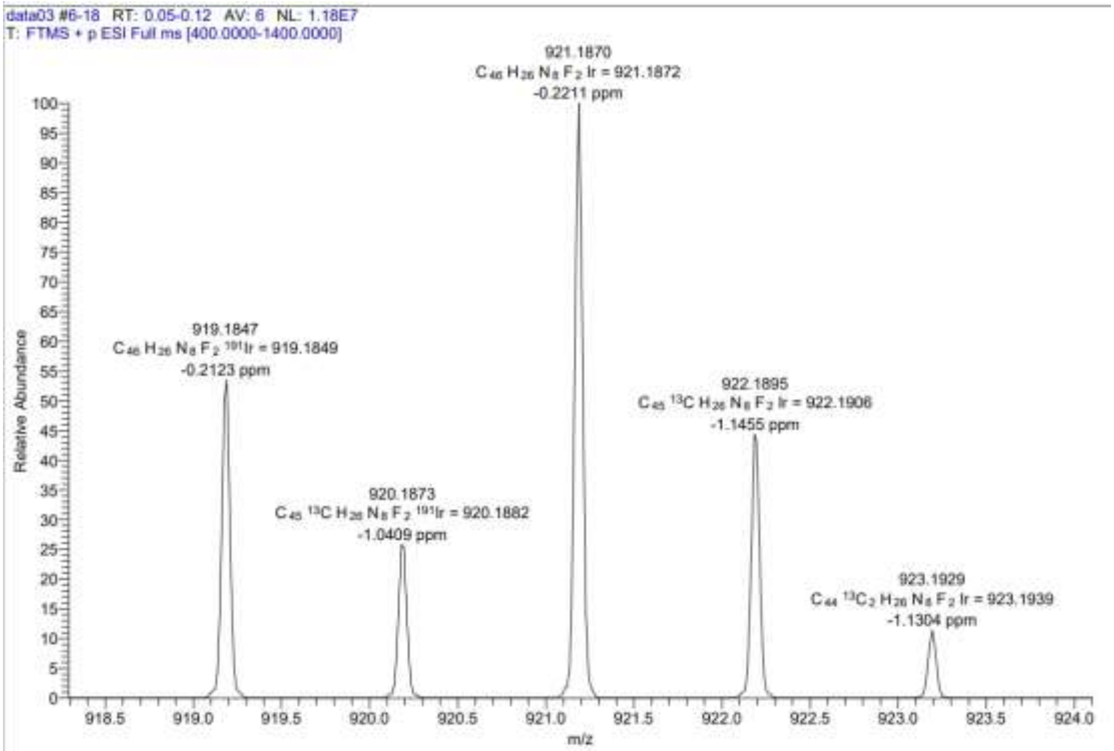

Figure S23. HRMS-ESI$^+$ spectrum of **FOMP**.

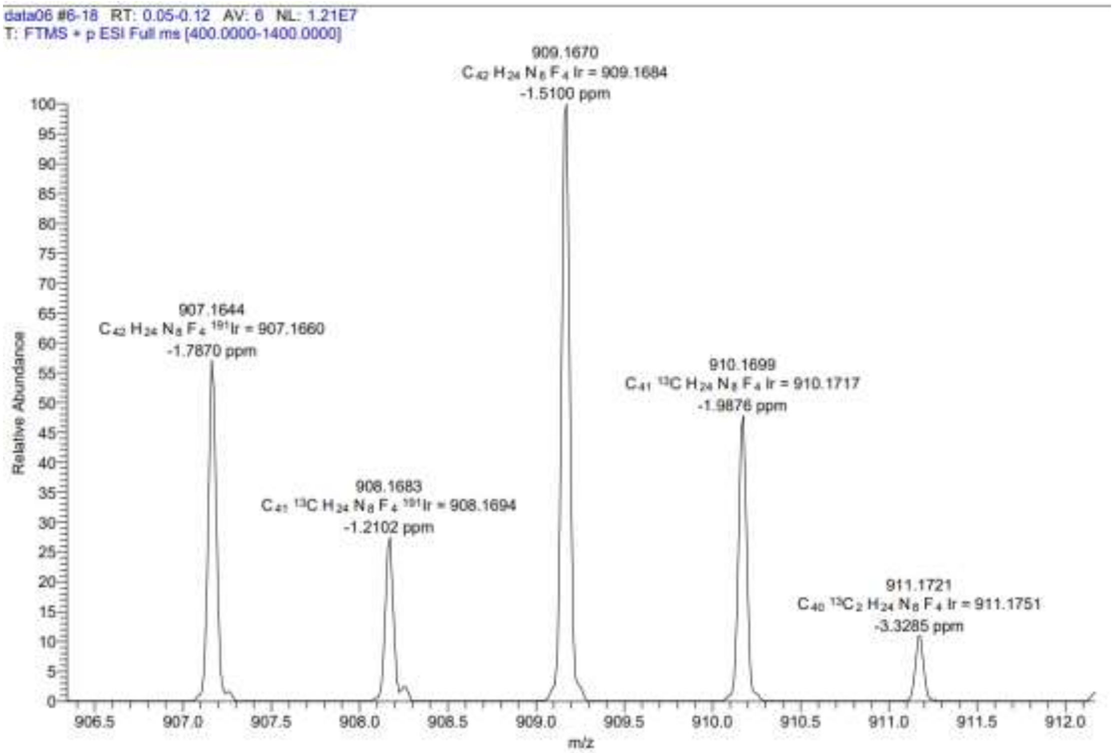

Figure S24. HRMS-ESI$^+$ spectrum of **DFBP**.

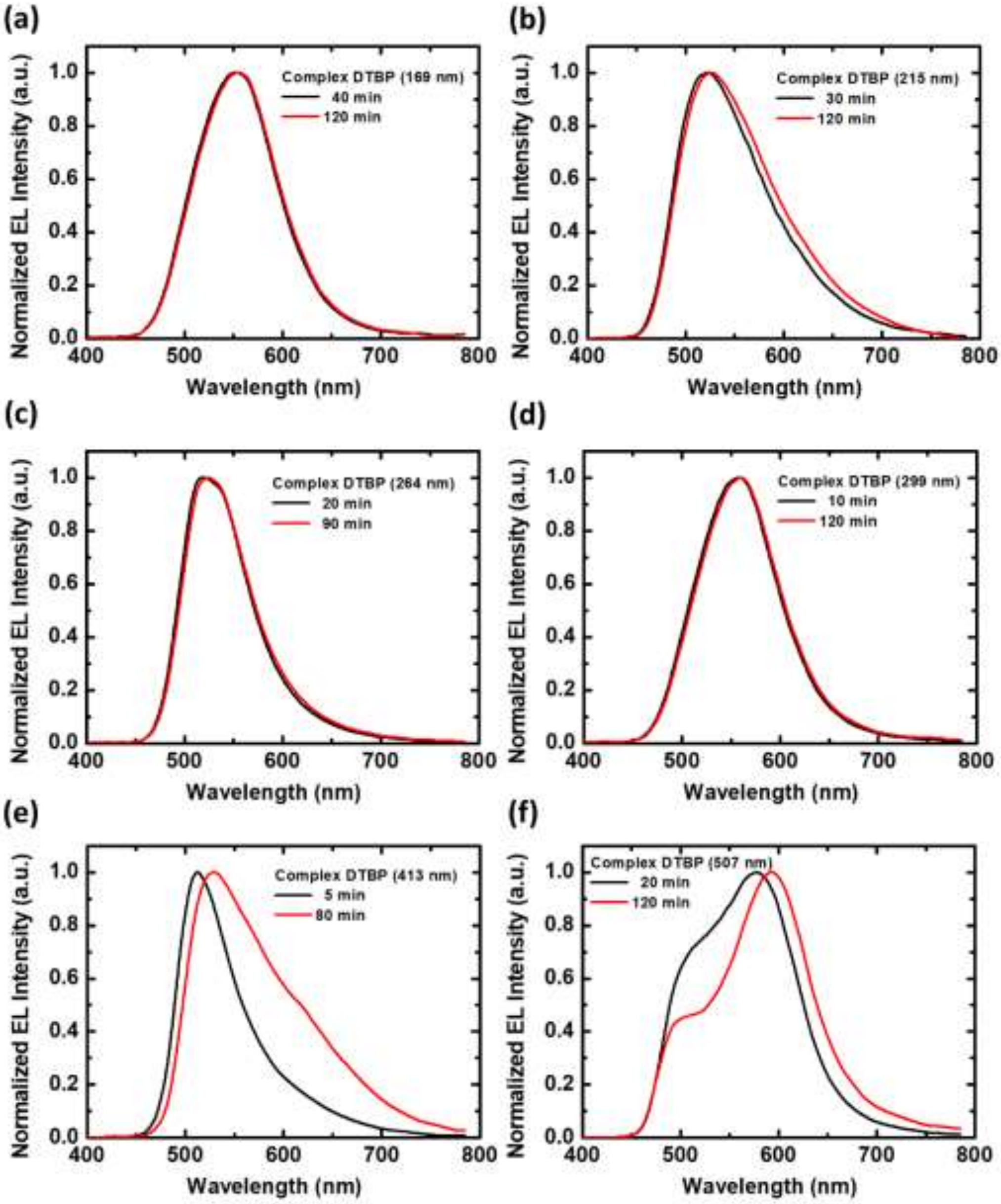

Figure S25. EL spectra of the LECs based on complex **DTBP** with emissive layer thickness (operating current) of a) 169 nm (0.25 μA), b) 215 nm (0.1 μA), c) 264 nm (0.25 μA), d) 299 nm (0.1 μA), e) 413 nm (0.1 μA), and f) 507 nm (0.1 μA).

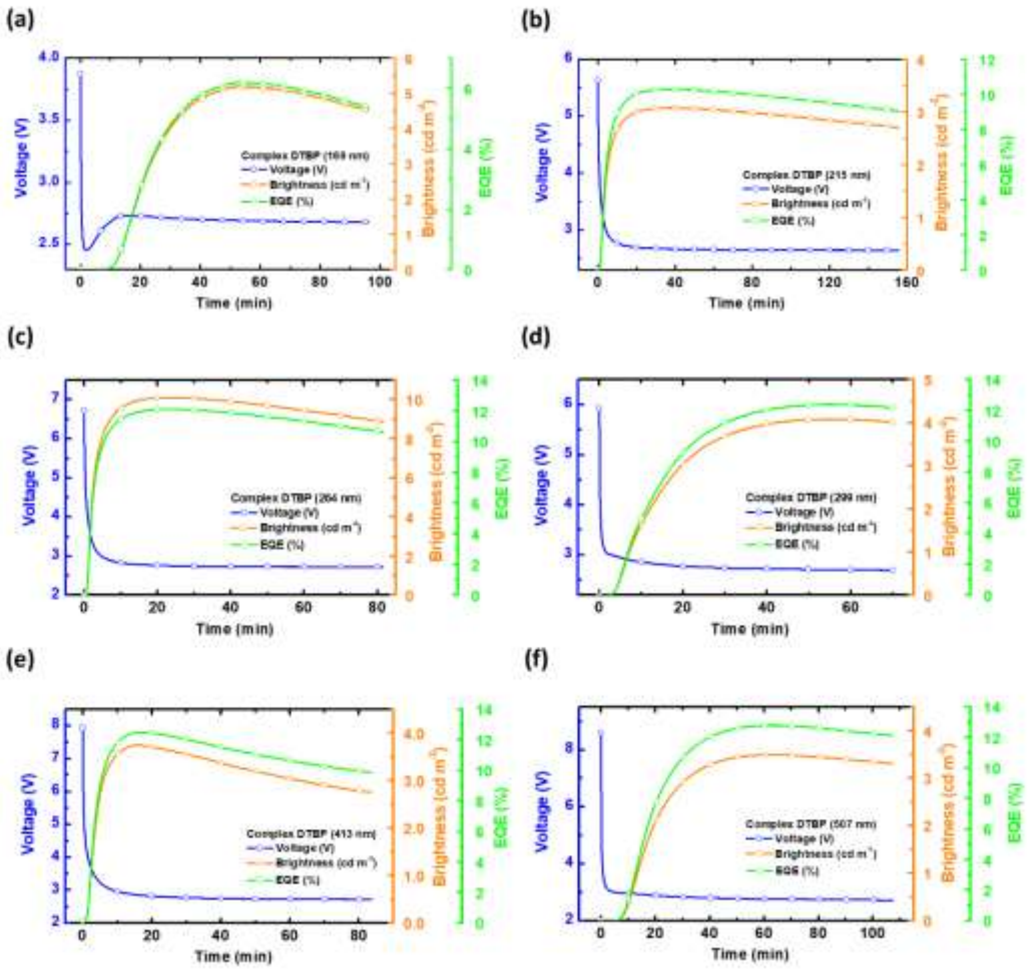

Figure S26. Time-dependent voltage (blue lines), brightness (orange lines), and EQE (green lines) of the LECs based on complex **DTBP** with emissive layer thickness (operating current) of a) 169 nm (0.25 μA), b) 215 nm (0.1 μA), c) 264 nm (0.25 μA), d) 299 nm (0.1 μA), e) 413 nm (0.1 μA), and f) 507 nm (0.1 μA).

References

(1) Ma, D.-L.; He, H.-Z.; Chan, D. S.-H.; Wong, C.-Y.; Leung, C.-H. A Colorimetric and Luminescent Dual-Modal Assay for Cu(II) Ion Detection Using an Iridium(III) Complex. *PLoS One* **2014**, *9*, e99930.
